# Volatility and Nonspecific van der Waals Interaction Properties of Per- and Polyfluoroalkyl Substances (PFAS): Evaluation Using Hexadecane/Air Partition Coefficients

**DOI:** 10.1021/acs.est.2c05804

**Published:** 2022-10-14

**Authors:** Jort Hammer, Satoshi Endo

**Affiliations:** Health and Environmental Risk Division, National Institute for Environmental Studies (NIES), Onogawa 16-2, 305-8506Tsukuba, Ibaraki, Japan

**Keywords:** per- and polyfluoroalkyl substance (PFAS), intermolecular interaction, linear free energy relationship (LFER), partition ratio, variable phase ratio headspace method, property estimation

## Abstract

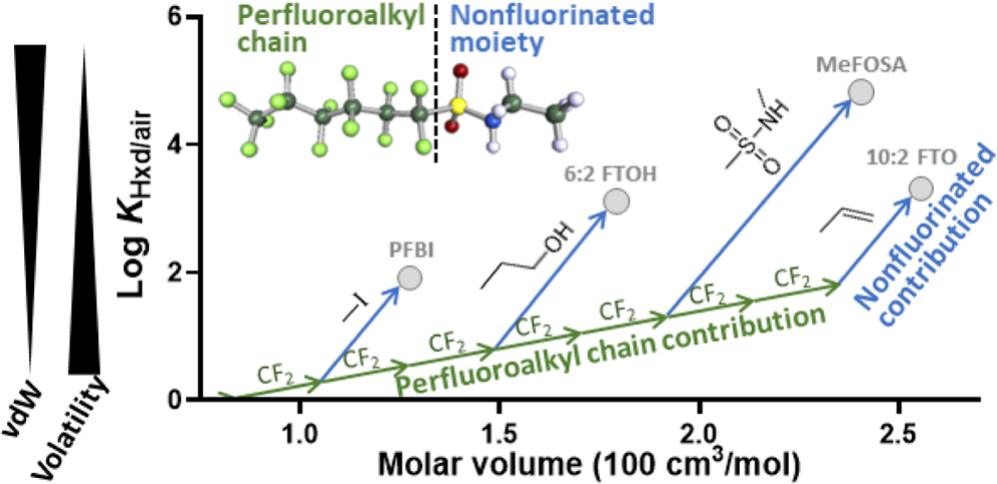

Per- and polyfluoroalkyl substances (PFAS) form weak van der Waals (vdW) interactions, which render this class of chemicals more volatile than nonfluorinated analogues. Here, the hexadecane/air partition coefficient (*K*_Hxd/air_) values at 25 °C, as an index of vdW interaction strength and volatility, were determined for 64 neutral PFAS using the variable phase ratio headspace and gas chromatographic retention methods. Log *K*_Hxd/air_ values increased linearly with increasing number of CF_2_ units, and the increase in log *K*_Hxd/air_ value per CF_2_ was smaller than that per CH_2_. Comparison of PFAS sharing the same perfluoroalkyl chain length but with different functional groups demonstrated that *K*_Hxd/air_ was highest for the *N*-alkyl perfluoroalkanesulfonamidethanols and lowest for the perfluoroalkanes and that the size of the nonfluorinated structure determines the difference in *K*_Hxd/air_ between PFAS groups. Two models, the quantum chemistry-based COSMO*therm* model and an iterative fragment selection quantitative structure–property relationship (IFS-QSPR) model, accurately predicted the log *K*_Hxd/air_ values of the PFAS with root-mean-square errors of 0.55 and 0.35, respectively. COSMO*therm* showed minor systematic errors for all PFAS, whereas IFS-QSPR exhibited large errors for a few PFAS groups that were outside the model applicability domain. The present data set will be useful as a benchmark of the volatilities of the various PFAS and for predicting other partition coefficient values of PFAS.

## Abstract 2

Hexadecane/air partition coefficient measurements quantitatively demonstrate the weak van der Waals interactions and high volatility of per- and polyfluoroalkyl substances.

## Introduction

Per- and polyfluoroalkyl substances (PFAS) are a large group of manufactured chemicals that have many industrial uses and are found in many everyday products. PFAS contain perfluorinated carbon atoms, which are highly resistant to degradation in the environment and ecosystems. Some PFAS such as perfluorooctane sulfonate and perfluorooctanoic acid have adverse health effects in humans,^1^ and many precursors exist that can transform into these or other potentially harmful PFAS.^2−5^ In 2018, the Organisation for Economic Co-operation and Development published a list of 4730 CAS-registered PFAS.^6^ Since then, the definition of PFAS has been expanded to include basically any compound that contains a perfluorinated methyl (−CF_3_) or methylene (−CF_2_−) moiety.^7^ To cope with the ever-increasing number of PFAS, many of which can be highly persistent, and avoid “regrettable substitutions”,^8^ it has been proposed that all PFAS should be regulated as a class.^9,10^ However, individual PFAS have different molecular properties and different transport and distribution properties in the environment, and therefore varying ecotoxicological relevance. Even if a class approach is implemented, large amounts of PFAS have been produced and persist in many products, wastes, landfills, and the environment;^11−13^ therefore, a greater understanding of the environmental fates and risks of individual PFAS is needed.^14^ Nevertheless, except for some acids of major concern and a few fluorotelomer alcohols, few experimental data describing the basic physicochemical properties of PFAS are available,^15^ which makes it difficult to perform reliable fate and risk assessments for diverse chemicals in the PFAS family.

PFAS are characterized by weak van der Waals (vdW) interaction properties.^16−18^ Therefore, PFAS form weak intermolecular interactions with their surrounding phase and tend to move into the atmosphere, unless strong specific interactions such as those between ionic PFAS and water take place. The hexadecane/air partition coefficient (*K*_Hxd/air_) is considered a quantitative metric of the vdW interaction properties of neutral solutes.^19,20^ For this reason, the logarithmic value of *K*_Hxd/air_ (also denoted as *L*) is used as a descriptor for the vdW interaction energy in polyparameter linear free energy relationships (pp-LFERs) such as linear solvation energy relationships (LSERs),^21,22^ which are used to predict partition coefficients for neutral organic chemicals. Thus, determining *K*_Hxd/air_ is an important step toward accurately predicting the partition coefficients of neutral PFAS in various phase partitioning systems. Along with saturated vapor pressure and octanol/air partition coefficient, *K*_Hxd/air_ is also an excellent indicator of volatility. Particularly, *K*_Hxd/air_ is an ideal surrogate parameter to evaluate the evaporation of a chemical from nonpolar phases such as polyethylene and polypropylene.^23,24^

The UFZ-LSER database currently contains *K*_Hxd/air_ values for more than 6400 neutral compounds.^25^ However, most of these compounds are hydrocarbon-based (i.e., nonfluorinated). *K*_Hxd/air_ values are available for only some short PFAS (C_≤3_) and a handful of other PFAS.^17^ This limited data availability stands in contrast to the extremely high interest in PFAS research and the importance of a greater understanding of the vdW interactions and volatility of these substances.

Here, we report new *K*_Hxd/air_ data for 64 neutral PFAS with various structural features obtained using two complementary approaches: the variable phase ratio headspace (VPR-HS) method and the gas chromatographic retention (GC-RT) method. The resulting data were compiled to form the first comprehensive data set of solvent/air partition coefficients for PFAS. This new data set allowed us to examine the structural features of PFAS that determine *K*_Hxd/air_ and therefore their vdW interaction properties and volatility. Finally, we used two computer models, a quantum chemistry-based prediction model (COSMO*therm*) and an iterative fragment selection quantitative structure–property relationship (IFS-QSPR) model, to predict *K*_Hxd/air_ values for the 64 PFAS. These prediction methods require only the molecular structure as the input parameter and could potentially be used to predict the *K*_Hxd/air_ values of PFAS that have not been determined experimentally.

## Materials and Methods

### Chemicals

*K*_Hxd/air_ values were determined for 64 PFAS, including perfluoroalkanes (PFAs), perfluoroalkyliodides (PFAIs), fluorotelomer alcohols (FTOHs), fluorotelomer iodides (FTIs), fluorotelomer olefins (FTOs), perfluoroalkane sulfonamides (PFASAs), *N*-alkylperfluoroalkanesulfonamides (FASAs), *N*-alkylperfluoroalkanesulfonamidoethanols (FASEs), fluoroethers (FEs), perfluorotrialkylamines (PFTAAs), fluorotelomer acrylates (FTACs), and fluorotelomer methacrylates (FTMACs). These chemicals were selected considering the environmental relevance, structural diversity, and availability. A complete list of the PFAS used is provided in Supporting Information (SI) Table S1 together with their abbreviations, CAS registry numbers, suppliers, and purities. Nonfluorinated chemicals used to relate GC retention times with *K*_Hxd/air_ values (hereafter referred to as reference chemicals) were obtained from various sources and are listed in the SI, Table S2. *n*-Hexadecane (anhydrous, ≥99%) was purchased from Sigma-Aldrich (Tokyo, Japan). Acetone and methanol were of analytical grade and obtained from Fujifilm Wako Chemicals (Osaka, Japan).

### VPR-HS Method

The VPR-HS method uses the mass balances within closed vials containing different volumes of an analyte solution;^26−28^ the method principle is described in detail in the cited articles. Briefly, in a closed system, a signal (*S*), such as GC peak area, obtained by headspace measurement follows eq 1
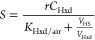
1where *r* is the response or proportionality factor between *S* and the concentration of the analyte in the headspace, *C*_Hxd_ is the original concentration of the analyte in the prepared hexadecane solution, and *V*_HS_/*V*_Hxd_ is the headspace-to-hexadecane solution volume ratio (*V*_HS_/*V*_Hxd_). Typically, the following linearized form of eq 1 is used for data fitting
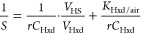
2Thus, 1/*S* is regressed against *V*_HS_/*V*_Hxd_ and the resulting intercept (*K*_Hxd/air_/*rC*_Hxd_) is divided by the slope (1/*rC*_Hxd_) to obtain *K*_Hxd/air_. However, in the present study, the original nonlinear form of the equation (i.e., eq 1) was used because the calculation could be completed in one step and it was straightforward to obtain the confidence interval (CI) of *K*_Hxd/air_. Here, fitting was performed with GraphPad Prism 9.3 using a weighting factor (1/*y*^2^) to obtain *K*_Hxd/air_ values and their 95% asymmetrical CI.

For each test chemical, the pure liquid or solid (ca 1 μL or 1 mg) was directly dissolved in 20 mL of hexadecane in a crimp-top glass vial closed with a PTFE-lined septum. No contamination signal from the PTFE was observed in GC measurements. The hexadecane solution was shaken for 24 h for dissolution, which was checked by eye. In case it was unclear whether complete dissolution was achieved, the supernatant was taken and diluted further with hexadecane by 20 times. The hexadecane solution was then quickly distributed into 20 mL glass headspace vials by a glass Pasteur pipette. The headspace vials were immediately closed with PTFE-lined septa, weighed, and stored in an incubator at 25 °C for at least 2 h. For each test chemical, five *V*_HS_/*V*_Hxd_ ratios were prepared in triplicate (i.e., 15 vials in total). The aimed *V*_HS_/*V*_Hxd_ ratios were between 0.5 and 250, depending on the expected *K*_Hxd/air_ of the test chemical based on the predicted values (see below). *V*_Hxd_ was calculated from the weight of hexadecane solution and the density of hexadecane. *V*_HS_ was obtained by subtracting the internal volume of the vials (22.16 ± 0.09 cm^3^, *n* = 10) with *V*_Hxd_. One hour before sampling, the vials were transferred to the GC autosampler tray, the temperature of which was kept at 25 °C by a Peltier Tray Cooler (Gerstel). The headspace in the vials was analyzed using a GC/MS system (7890A GC/5975C MS, Agilent Technologies; MPS2 autosampler, Gerstel). The analytical conditions are described in SI-1. Response linearity was confirmed in preliminary tests.

### GC-RT Method

Two nonpolar capillary columns were used: a squalane (SQ) column (CP-Squalane, 100 m × 0.25 mm i.d., 0.20 μm coating thickness, Agilent Technologies) and a poly(50% methyl–50% octylsiloxane) column (SPB-Octyl, 30 m × 0.25 mm i.d., 0.25 μm coating thickness, Supelco). SQ is a C_30_ hydrocarbon liquid (2,6,10,15,19,23-hexamethyltetracosane) and has long been used as a nonpolar reference phase for GC.^29^ Since SQ and hexadecane are both alkanes, no specific molecular interactions occur in either phase, and *K*_Hxd/air_ can be determined from retention time in the SQ column.^30,31^ Several disadvantages of SQ for GC column use such as high volatility, instability, and impurity have been noted.^30,32^ Particularly, the low maximal operating temperature (90 °C for CP-Squalane) makes SQ columns only applicable to relatively volatile chemicals. In contrast, the SPB-Octyl column has a siloxane-based structure and therefore has a higher maximal operating temperature (280 °C) compared with the CP-Squalane column. SPB-Octyl is less polar than commonly used poly(dimethylsiloxane) columns (e.g., HP-1) and has been used previously for the determination of *K*_Hxd/air_ values.^33,34^ However, weak polar interactions could arise due to the presence of the siloxane structure,^35^ the significance of which must be evaluated.

For retention time measurement, 10 and 75 m columns were cut from the original 100 m CP-Squalane column. The outlet of each column was connected to a 25 cm piece of a deactivated fused silica capillary column (0.1 mm i.d., GL sciences), which served as a restrictor to maintain the column internal pressure and as a transfer line to the MS ionization chamber heated at 230 °C and acted to reduce column bleeding. The SPB-Octyl column was used as is. Retention time measurements on the 75 and 10 m SQ columns were performed at an isothermal oven setting of 30 °C, and on the SPB-Octyl column at 30, 70, or 100 °C. The chemicals were introduced into the column by injection of 250–2500 μL of the headspace above the pure liquid or solid of a test chemical. For retention time measurement at 70 and 100 °C with the SPB-Octyl column, 1 μL of acetone solution was injected into the GC for chemicals that were not volatile enough for headspace injection. The headspace and liquid injection methods were compared in preliminary tests and resulted in no difference (data not shown). Further details of the GC-RT measurement approach are provided in SI-2.

Capacity factor (*k′*) was calculated using the retention time of the chemical (*t*) and that of an unretained tracer compound (*t*_0_)
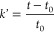
3For determination of *t*_0_, Ar in air was monitored at an *m*/*z* of 40. Data for PFAS with *t* – *t*_0_ < 0.1 min were not considered. For reference chemicals, an even stricter criterion (*t* – *t*_0_ < 0.3 min) was applied to extract data of only the highest accuracy.

Values of *k′* for reference chemicals with known *K*_Hxd/air_ values (Table S2, values obtained from the UFZ-LSER database)^25^ were measured and the following equation was fitted to the data
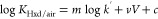
4where *V* is McGowan’s molar volume (in units of 10^2^ cm^3^ mol^–1^, as used in the LSER model),^21^*m* and *v* are the fitting coefficients, and *c* is the fitting constant. Because *V* can be calculated from the molecular structure,^36^ log *K*_Hxd/air_ for a given PFAS can be obtained with the measured value of log *k*′. Note that, when calculating *V*, we used a refined incremental value of 12.48 cm^3^ mol^–1^ for the atom-specific volume of F, as described in ref (17).

### Prediction by COSMO*therm* and IFS-QSPR

The COSMO*therm* algorithm uses COSMO-RS theory and can calculate the free energy of solvation in hexadecane and log *K*_Hxd/air_ value of a compound.^37^ In this approach, the surface polarities on the molecules are obtained by quantum chemical calculations, and pairwise interaction energy between surface segments of solute and solvent molecules is used in statistical thermodynamic calculations to derive the solvation free energy of solute in solvent. COSMO*therm* has been used to predict log *K*_Hxd/air_ values for a number of environmentally relevant chemicals including pesticides, flame retardants, and hormones.^33,34^ The prediction errors reported in these previous studies are within ±1 log unit in most cases, even for complex, multifunctional compounds. Although COSMO*therm* has yet to be used to predict the log *K*_Hxd/air_ values of PFAS, it has been used to predict other partition coefficients of PFAS; however, these predictions were compared to experimental data only for FTOHs, FTOs, and a few other PFAS because of limited data availability.^38,39^ In the present study, quantum chemical calculations and conformer selection were performed using Turbomole and COSMO*confX* (both version 2021, COSMO*logic*, Dassault Systèmes), respectively, which yielded a complete set of relevant conformations with full geometry optimization in the gas phase and in the conductor reference state. The resulting set of quantum chemical calculations for conformers were then transferred to the COSMO*thermX* software (version 2021, COSMO*logic*, Dassault Systèmes) to calculate log *K*_Hxd/air_ at 25 °C using the COSMO*therm* algorithm (parameterization, BP_TZVPD_FINE_21).

Recently, Brown^40^ developed a quantitative structure–property relationship (QSPR) for log *K*_Hxd/air_ that uses an iterative fragment selection (IFS) algorithm. The IFS method automatically selects two-dimensional molecular fragments that are significant for the property of interest and generates a group contribution–based QSPR model. The published IFS-QSPR model for log *K*_Hxd/air_ demonstrated high prediction accuracy (0.286 log units RMSE) in external validation.^40^ The latest version of IFS-QSPR for log *K*_Hxd/air_^41^ is implemented in EAS-E Suite Ver. 0.95 2022,^42^ which is available free of charge on the Internet (www.eas-e-suite.com), and was used here to predict log *K*_Hxd/air_ values.

## Results and Discussion

### *K*_Hxd/air_ Values Determined by the VPR-HS Method

*K*_Hxd/air_ values at 25 °C were measured for 16 PFAS by the VPR-HS method (Table 1). Plots of GC peak area versus phase ratio (*V*_HS_/*V*_Hxd_) are shown in Figure S1. The 95% CIs were 88–115% of the determined *K*_Hxd/air_ values on average and 79–133% in the worst case (Table S3). On the log scale, these CIs corresponded to ±0.06 and ±0.11 log units, respectively. The log-converted *K*_Hxd/air_ values were in the range of 1.33–2.69. For compounds with higher *K*_Hxd/air_ values, it was difficult to measure significant changes in GC peak area at different *V*_HS_/*V*_Hxd_ ratios using the current setup with 20 mL vials. Lei et al.^28^ reported that the VPR-HS method is suitable for measuring solvent/air partition coefficients that are <3.3 log units. This upper limit is somewhat higher than ours, which can be explained by the fact that Lei et al.^28^ applied phase ratios up to 500 in comparison to 250 in the present study. In the literature, Goss et al.^17^ have reported a log *K*_Hxd/air_ value of 1.35 for 4:2 FTO, which is the only log *K*_Hxd/air_ value determined for PFAS by VPR-HS so far and is comparable with the value obtained in the present study (1.44). Generally, the VPR-HS method is a direct measurement method with few assumptions;^26−28^ therefore, the *K*_Hxd/air_ values obtained using this method are considered highly reliable.

**Table 1:** Log *K*_Hxd/air_ Values (25 °C) Determined in the Present Study^a^

compound	VPR-HS	SQ 75 m,30 °C	SQ 10 m, 30 °C	SPB-Octyl,30 °C	SPB-Octyl,70 °C	SPB-Octyl,100 °C	recommended value	literature
3:1 FTOH	1.54			1.77			1.54	1.94^b^
3:3 FTOH	2.69			2.88	2.85		2.69	
4:2 FTOH	2.37			2.57			2.37	
4:4 FTOH					3.63	3.52	3.57	
6:2 FTOH				3.14	3.13		3.14	3.38^b^, 2.96^c^
7:1 FTOH				2.96			2.96	3.01^b^
8:2 FTOH					3.69	3.60	3.64	3.47^c^
10:2 FTOH					4.25	4.22	4.23	3.90^c^
12:2 FTOH					4.80	4.83	4.82	
5:2s FTOH	2.16			2.63			2.16	
NFHp-1,2-diol					3.60	3.48	3.54	
PFPrAnhy	1.62	0.99					1.62	
EtFHxSE						5.78	5.78	
EtFOSE						6.37	6.37	
MeFBSE						4.84	4.84	
MeFOSE						6.02	6.02	
PFBSA					3.87	3.61	3.74	
PFHxSA					4.44	4.23	4.34	
PFOSA						4.84	4.84	
MeFBSA					3.75	3.59	3.67	
MeFHxSA					4.30	4.21	4.26	
MeFOSA					4.86	4.82	4.84	
EtFHxSA					4.55	4.49	4.52	
EtFOSA					5.10	5.10	5.10	
PFBSF		1.29					1.29	
PFBI	1.93	1.95	1.90	1.89			1.93	
PFHxI		2.50	2.44	2.48			2.48	
PFHpI		2.76	2.69	2.76	2.89		2.78	
PFOI		3.03	2.99	3.04	3.15		3.05	
PFDI		3.57	3.53	3.60	3.71	3.69	3.62	
1,8-DIPFO						5.15	5.15	
4:2 FTI		3.34	3.30	3.32	3.37	3.29	3.32	
6:1 FTI		3.41	3.34	3.38			3.38	
6:1 FTI-7H			3.87		3.97	3.93	3.93	
6:2 FTI			3.85	3.87	3.94	3.92	3.90	
8:2 FTI			4.40		4.48	4.51	4.47	
10:2 FTI					5.04	5.12	5.08	
FE-E3	1.78	1.98	1.88	1.93			1.78	
FE-E4	2.34	2.68	2.37	2.75			2.34	
FE-E5	2.66	3.42		3.56			2.66	
AFOE		3.61	3.52	3.58			3.57	
FE-E1-I	1.97	1.91		1.90			1.97	
APFIPE	1.74	1.73		1.66			1.74	
PFTPrA		1.52		1.45			1.48	
PFTBA		2.28		2.27			2.27	
PFHp		1.07					1.07	1.65^b^, 1.12^d^
PFO	1.33	1.35					1.33	2.17^b^
PFN	1.56	1.55					1.56	2.64^b^, 1.77^d^
PFDoD		2.39	2.14	2.37			2.30	
1,8-DHPFO		2.23	2.06	2.20			2.16	
1,8-DVPFO			3.84	3.95	4.03	4.00	3.95	
PFOSt					5.62	5.73	5.67	
4:2 FTO	1.44	1.44		1.45			1.44	1.35^c^
6:2 FTO	2.05	1.99	2.02	1.96			2.05	1.83^c^
8:2 FTO	2.42	2.52	2.49	2.53			2.42	2.31^c^
6:2 FTAC					4.17	4.13	4.15	
8:2 FTAC					4.72	4.74	4.73	
10:2 FTAC					5.27	5.34	5.30	
4:2 FTMAC					4.06	3.99	4.03	
6:2 FTMAC					4.61	4.60	4.60	
8:2 FTMAC					5.15	5.21	5.18	
10:2 FTMAC					5.70	5.80	5.75	
6:2 FTBnOH						6.72	6.72	
8:2 FTAce					4.32	4.30	4.31	

a Abbreviations are also explained in Table S1, Supporting Information, with the CAS registry numbers.

b Abraham’s Absolv data retrieved from UFZ-LSER database.^25^

c Ref (17).

d Ref (19). NFHp-1,2-diol, 3-(perfluoro-2-butyl)propane-1,2-diol; PFPrAnhy, pentafluoropropanoic anhydride; EtFHxSE, *N*-ethyl perfluorohexane sulfonamidoethanol; EtFOSE, *N*-ethyl perfluorooctane sulfonamidoethanol; MeFBSE, *N*-methyl perfluorobutane sulfonamidoethanol; MeFOSE, *N*-methyl perfluorooctane sulfonamidoethanol; PFBSA, perfluorobutane sulfonamide; PFHxSA, perfluorohexane sulfonamide; PFOSA, perfluorooctane sulfonamide; MeFBSA, *N*-methyl perfluorobutane sulfonamide; MeFHxSA, *N*-methyl perfluorohexane sulfonamide; MeFOSA, *N*-methyl perfluorooctane sulfonamide; EtFHxSA, *N*-ethyl perfluorohexane sulfonamide; EtFOSA, *N*-ethyl perfluorooctane sulfonamide; PFBSF, perfluorobutanesulfonyl fluoride; PFBI, perfluorobutyl iodide; PFHxI, perfluorohexyl iodide; PFHpI, perfluoroheptyl iodide; PFOI, perfluorooctyl iodide; PFDI, perfluorodecyl iodide; 1,8-DIPFO, 1,8-diiodoperfluorooctane; AFOE, allyl 1H,1H-perfluorooctyl ether; FE-E1-I, 1-(heptafluoropropoxy)-1,2,2,2-tetrafluoro-1-iodoethane; APFIPE, allyl perfluoroisopropyl ether; PFTPrA, perfluorotripropyl amine; PFTBA, perfluorotributyl amine; PFHp, perfluoroheptane; PFO, perfluorooctane; PFN, perfluorononane; PFDoD, perfluorododecane; 1,8-DHPFO, 1H,8H-perfluorooctane; 1,8-DVPFO, 1,8-divinylperfluorooctane; PFOSt, 4-(perfluorooct-1-yl)styrene; 6:2 FTBnOH, 4-(3,3,4,4,5,5,6,6,7,7,8,8,8-tridecafluorooctyl)benzyl alcohol; 8:2 FTAce, 1H,1H,2H,2H-perfluorodecyl acetate.

### *K*_Hxd/air_ Values Determined by the GC-RT Method

We measured log *k*′ values for 11 reference chemicals and 25 PFAS on the 75 m SQ column, and for 9 reference chemicals and 18 PFAS on the 10 m SQ column (Table S4). However, chemicals with polar functional groups (e.g., −C=O, −OH) showed peaks with extensive tailing and their *k*′ values could not be determined with either of the SQ columns. In contrast, SPB-Octyl is inert and this column could be used to determine *k*′ values for polar chemicals; consequently, log *k*′ values at 30, 70, and 100 °C were obtained for 26, 29, and 39 reference chemicals, and 28, 32, and 35 PFAS, respectively.

In most cases, there was only one peak in the chromatogram that was distinctly higher than the others. However, the chromatograms for FE-E3, FE-E4, and FE-E5 showed 2, 3, and 7 peaks, respectively, when the SQ columns were used, and 1, 3, and 7 peaks, respectively, when the SPB-Octyl column was used (Figure S2), suggesting the presence of isomers. Nevertheless, the retention times of these isomers were within narrow ranges that corresponded to 0.01, 0.04, and 0.07 log units of *K*_Hxd/air_ for FE-E3, FE-E4, and FE-E5, respectively (see Table S4 for individual data). For simplicity in the following discussion, the log *k*′ values that corresponded to the first peak in the chromatogram for FE-E3 and the middle peaks in the chromatograms for FE-E4 and FE-E5 are used to represent all isomers of those FEs.

Initially, eq 4 was fitted only to the log *k*′ data for the reference chemicals (Table S5). Among the reference chemicals, 2,2-dimethylpentane in the data set obtained using the 75 m SQ column and dodecamethylcyclohexasiloxane in the data set obtained using the SPB-Octyl column (100 °C) were removed as statistical outliers (>3SD). The log *k*′ and *V* values for each PFAS were inserted into the calibrated equations and the log *K*_Hxd/air_ value was calculated. The log *K*_Hxd/air_ values obtained this way were systematically higher than those determined by the VPR-HS method (Figure S3). The mean difference was 0.33 ± 0.40 log units, and the difference was particularly large (0.56–1.36 log units) for the three FEs (i.e., FE-E3, FE-E4, and FE-E5). Such systematic measurement bias has not been reported for either of the methods.^26,28,30,31,33,34^ Possible contributions of polar interactions on log *k*′ were evaluated and found to be small or not present (see SI-3, Table S6). A contributing reason for these discrepancies was that the reference chemicals used for calibration of eq 4 did not include any PFAS. Plotting log *k*′ versus *V* (Figure S4) revealed that the reference chemicals and PFAS occupied different spaces within the two-dimensional plot area. Hence, the equation calibrated using only the hydrocarbon-based reference chemicals must be extrapolated to obtain the log *K*_Hxd/air_ values for the PFAS, resulting in a relatively large error in the determined log *K*_Hxd/air_ value.

To minimize the error introduced due to extrapolation, PFAS with *K*_Hxd/air_ values measured by VPR-HS were added to the calibration set used to calibrate eq 4. The three FEs were not included in the calibration set because of the presence of multiple peaks, as discussed earlier in this section. Pentafluoropropanoic anhydride (PFPrAnhy) was an outlier (>3SD) in the SQ column data sets and 5:2s FTOH was an outlier in the SPB-Octyl column data sets when included in the calibration and therefore these PFAS were also excluded. In the end, 18 and 12 chemicals for the 75 and 10 m SQ column, respectively, and 35, 30, and 39 chemicals for the SPB-Octyl column at 30, 70, and 100 °C, respectively, were used for calibration of eq 4. The inclusion of PFAS in the calibration of eq 4 resulted in lower *R*^2^ and higher SD values, whereas the standard errors for the fitting coefficients were comparable to or even lower than those obtained without considering the PFAS in the calibration (Tables 2 and S5). The measured and fitted log *k*′ values are compared in Figure S5. With these updated equations, *K*_Hxd/air_ values for the rest of the PFAS were determined (Table 1; see Table S7 for prediction intervals in each data). Note that, for SPB-Octyl at 70 and 100 °C, data for only one and zero PFAS, respectively, were available for calibration and therefore the equations virtually could not be improved. Nevertheless, the 70 and 100 °C SPB-Octyl data sets contain two and three methylsiloxanes, respectively, which, like PFAS, also have weak vdW interaction properties and fall in a similar space within the log *k*′–*V* diagram (Figure S4).

**Table 2:** Fitting Coefficients of Equation 4 Calibrated Using Data for Reference Chemicals and Selected PFAS^a^

	*m*	*v*	*c*	*R*^2^	SD	*n*
CP-squalane75 m,30 °C	1.058	–0.050	2.658	0.994	0.044	18
	(0.021)	(0.020)	(0.026)			
CP-squalane10 m,30 °C	1.104	–0.050	2.565	0.997	0.054	12
	(0.020)	(0.037)	(0.048)			
SPB-octyl, 30 °C	1.112	0.057	2.586	0.986	0.090	35
	(0.023)	(0.034)	(0.038)			
SPB-octyl,70 °C	1.269	0.247	3.197	0.994	0.060	30
	(0.021)	(0.030)	(0.032)			
SPB-octyl,100 °C	1.540	0.396	3.585	0.991	0.093	39
	(0.027)	(0.032)	(0.044)			

a Values in parentheses are the standard errors.

Overall, log *K*_Hxd/air_ values for 64 PFAS were determined by GC-RT. The *K*_Hxd/air_ values obtained under the different measurement conditions agreed well with one another (Table 1 and Figure S6). The standard deviation of log *K*_Hxd/air_ measured by GC-RT was 0.06 log units on average, and 0.20 (FE-E4) in the worst case. Agreement of log *K*_Hxd/air_ values between the VPR-HS and GC-RT methods was high overall (mean difference, 0.05 ± 0.27) and also high for the three FEs (difference, 0.15–0.91), which was expected because the VPR-HS data were included in the calibration data sets. A recommended value of log *K*_Hxd/air_ for each PFAS is given in Table 1. As discussed in the previous section, data determined by VPR-HS are considered to be highly reliable. In the absence of VPR-HS data, the mean of the log *K*_Hxd/air_ values determined by GC-RT were chosen instead. The log *K*_Hxd/air_ values determined in the present study agree well with the available literature data (see Table 1).

### PFAS Structure and Log *K*_Hxd/air_ Value

**Figure 1:**
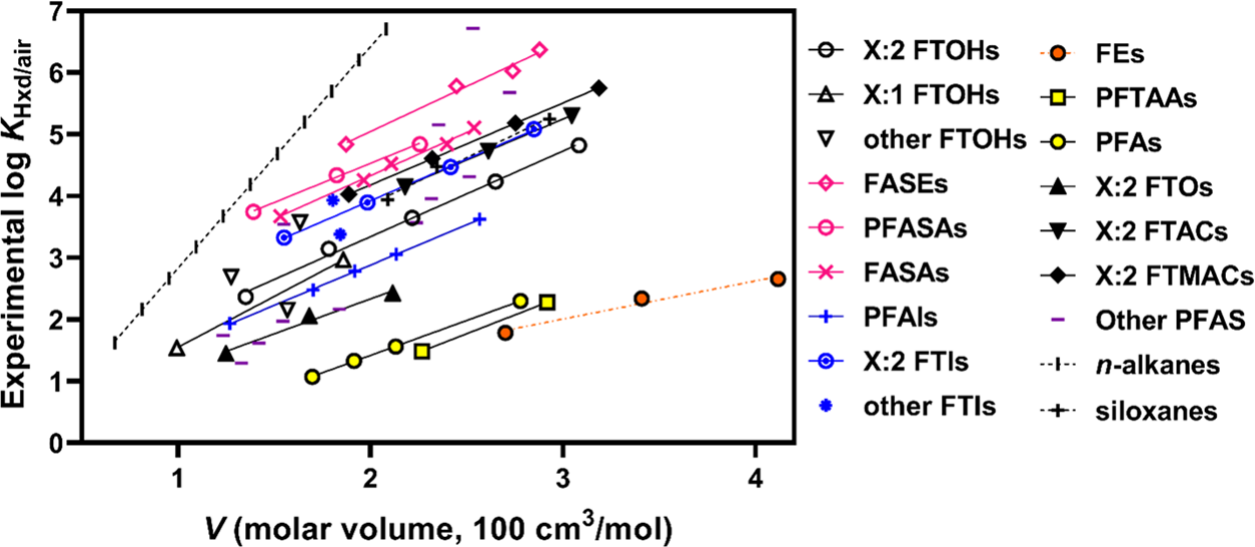
Experimentally determined log *K*_Hxd/air_ values for PFAS versus McGowan’s molar volume (*V*). Lines indicate the linear regression for each group of compounds sharing the same functional end group.

To explore the size dependence of log *K*_Hxd/air_, the obtained experimental log *K*_Hxd/air_ values were plotted against molar volume (*V*) (Figure 1). Within each PFAS group, log *K*_Hxd/air_ increased linearly with increasing length of the perfluoroalkyl chain, and the slope was comparable for all of the groups (except for FEs). This result indicates that the CF_2_ increment has the same contribution to log *K*_Hxd/air_ irrespective of what functional group the molecule may contain. The slope for the perfluoroalkyl chain (0.0130 ± 0.0007 mol/cm^3^) was substantially lower than that for the hydrogenated alkyl chain (0.0360 mol/cm^3^, as illustrated by *n*-alkanes in Figure 1). These slopes correspond to an increase of 0.28 log units per CF_2_ and 0.51 log units per CH_2_, demonstrating quantitatively the weak vdW interaction property of the perfluorinated alkyl structure. In FEs, the repeating unit is not CF_2_ but a branched perfluoroalkyl ether group (–O–CF(CF_3_)–CF_2_−), which could be a reason for the slope of FEs being more gentle than that of the other groups. However, the data difference between VPR-HS and GC-RT was relatively large for FEs, as shown above, suggesting further scrutiny needed to draw a definitive conclusion.

**Figure 2:**
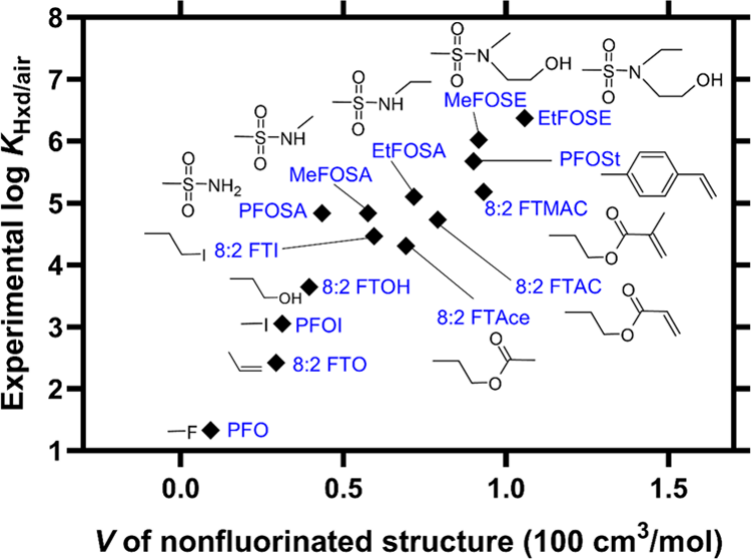
Experimentally determined log *K*_Hxd/air_ values for the PFAS with a perfluorooctyl chain (−C_8_F_17_) versus McGowan’s molar volume (*V*) subtracted by the contribution of −C_8_F_18_ (182 cm^3^/mol).

For a given value of *V* (i.e., size of the molecule), the highest and lowest *K*_Hxd/air_ values differed by about 4 log units. The highest *K*_Hxd/air_ values were observed for the FASEs, followed by the PFASAs and FASAs, all of which contain the bulky sulfonamide group. The lowest *K*_Hxd/air_ values were observed for the PFTAAs and PFAs, which have no or only a small nonfluorinated structure. These results suggest that the size of the nonfluorinated part of the molecule explains the difference in *K*_Hxd/air_ between PFAS groups. The influence of the nonfluorinated part of the molecule on *K*_Hxd/air_ was even clearer when the 14 PFAS that contain a perfluorooctyl group (−C_8_F_17_) were extracted and compared (Figure 2). The log *K*_Hxd/air_ values for these 14 PFAS spanned 5 log units, and the values correlated positively with the size of the nonfluorinated part (*R*^2^, 0.85).

### Prediction of log *K*_Hxd/air_ by COSMO*therm* and IFS-QSPR

**Figure 3:**
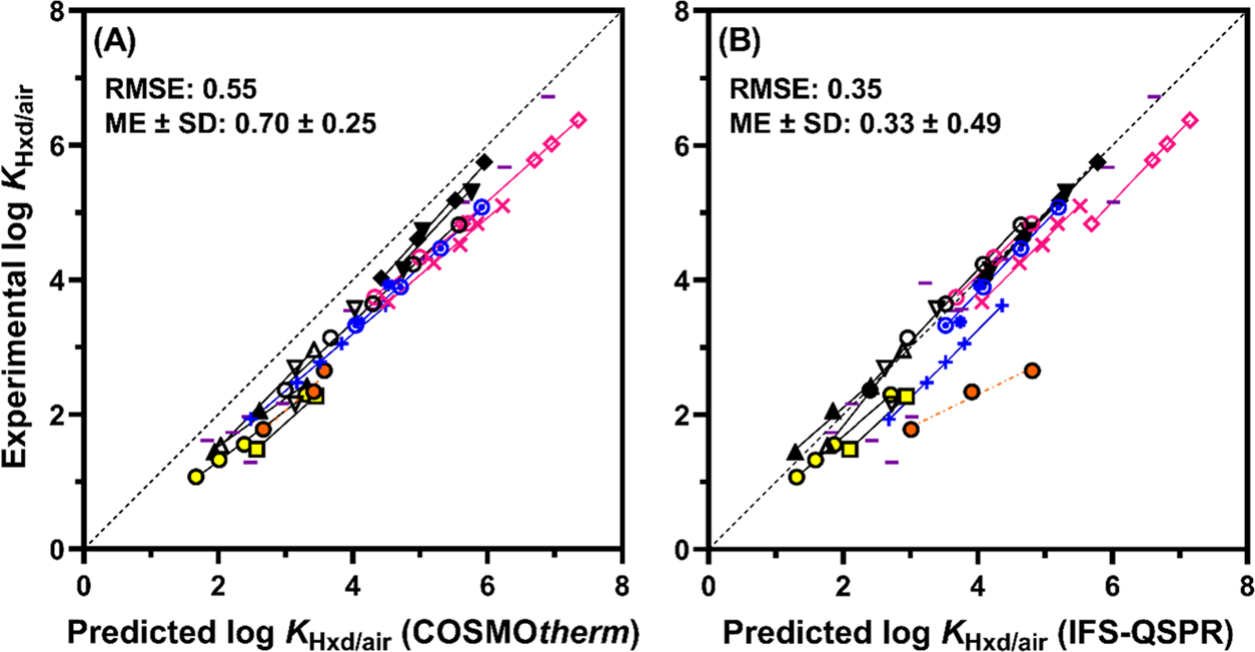
Experimental log *K*_Hxd/air_ values versus values predicted by COSMO*therm* (A) or IFS-QSPR (B). Experimental values are the “recommended values” in Table 1. Symbols are explained in Figure 1. RMSE, root-mean-square error; ME, mean of prediction errors; SD, standard deviation of prediction errors. Solid lines indicate the linear regression for each group, and the dashed line indicates the 1:1 relationship.

Log *K*_Hxd/air_ values for the 64 PFAS were predicted using COSMO*therm* and IFS-QSPR (Figure 3; all values are presented in Table S8). The predictions by both models agreed well with the experimental values, with root-mean-square errors (RMSEs) of 0.55 and 0.35, respectively. However, there were some differences between the results of the two models.

The predictions by COSMO*therm* showed excellent correlation with the experimental data (*R*^2^, 0.97). However, COSMO*therm* systematically overestimated log *K*_Hxd/air_, as indicated by a positive mean error of 0.70. Such a systematic prediction bias did not occur for hydrocarbon-based compounds (Figure S7). The PFAS groups with increasing perfluoroalkyl chain length were aligned parallel to the 1:1 line, with a slightly larger prediction error for the longer-chain PFAS. For example, the prediction error for perfluorobutyl iodide (PFBI) was +0.55 log units, whereas that for perfluorodecyl iodide (PFDI) was +0.87 log units. These results show that the current version of the COSMO*therm* algorithm largely captures but slightly overestimates the nonspecific vdW interaction properties of PFAS and that it may underestimate the volatility of PFAS with a long perfluorinated alkyl structure. Noteworthily, two previous studies^43,44^ reported that COSMO*mic*, a submodule of COSMO*thermX* software, substantially underpredicted the perfluoroalkyl chain length dependence of phospholipid membrane/water partition coefficients for perfluoroalkyl acid anions. The present study demonstrated that COSMO*therm* can at least predict the vdW interactions of neutral PFAS in a homogeneous phase.

IFS-QSPR produced a smaller RMSE (0.35) and a smaller mean error (0.33 ± 0.49) than COSMO*therm*. Prediction errors by IFS-QSPR were within ±0.3 log units for 36 of the 64 PFAS, compared to only 3 PFAS by COSMO*therm*. Thus, the predictions by IFS-QSPR, on average, were more accurate than those of COSMO*therm* (Figure 3). However, the plot of the IFS-QSPR predictions showed greater scattering of the data points because of the relatively large prediction errors for some PFAS, e.g., FASEs (prediction error, 0.78–0.85), PFAIs (0.74–0.76), and perfluorobutanesulfonyl fluoride (PFBSF) (1.43). Predictions for the three FEs particularly deviated from the experimental data (prediction error, 1.23–2.16). Because IFS-QSPR is principally an empirical fit model, prediction accuracy depends strongly on the training data set, which suggests that the structures of the PFAS groups examined here may not be accurately represented by the training set used for model calibration. In the plot of experimental versus predicted log *K*_Hxd/air_ values (Figure 3), the data points for the different groups of PFAS are parallel to the 1:1 line, indicating that the contribution of the CF_2_ unit to the value of log *K*_Hxd/air_ is accurately captured by the model. Therefore, the observed large prediction errors for some PFAS are most likely the result of insufficient calibration of the model for the nonfluorinated substructure. The EAS-E Suite software also calculates the uncertainty level (UL) of IFS-QSPR predictions for log *K*_Hxd/air_ based on multiple indicators including the leverage and the structure similarity to indicate whether the prediction made is within the applicability domain of the model. Out of the 64 PFAS, 30, 27, and 7 PFAS were labeled with a UL of 0, 1, and 2, respectively (Table S8). The RMSE value for the PFAS labeled with a UL of 0, 1, and 2 was 0.19, 0.27, and 1.29, respectively (Figure S8). Thus, IFS-QSPR can provide reliable predictions for PFAS with a UL of 0 or 1, but substantially less reliable predictions for PFAS with a UL of 2. The UL provided by EAS-E Suite appears to be highly useful to judge the accuracy of the model prediction and indicates that many of the PFAS used in this work are within the applicability domain (UL 0 or 1) of the current version of IFS-QSPR for log *K*_Hxd/air_.

As an additional comparison, an earlier version of IFS-QSPR implemented at the website of the UFZ-LSER database was also used to predict log *K*_Hxd/air_ values for the 64 PFAS. The prediction gave similar statistics (RMSE, 0.33; mean error ± SD, 0.32 ± 0.48) compared to the latest version, but the error for each compound was often substantially different (Figure S9). In particular, the CF_2_ increment was not correctly captured by the former version. The latest version is based on an updated training data set and fragment pool,^41^ and the present results suggest that the version update improved predictions for PFAS.

### Implications and Recommendations

Here, we determined experimental log *K*_Hxd/air_ values for a large set of neutral PFAS to clarify their volatility from nonpolar phases and their nonspecific vdW interaction properties. The data indicate that the PFAS examined here are generally volatile, and that the nonfluorinated part of the PFAS molecule has a large influence on log *K*_Hxd/air_ value. PFAS with a small nonfluorinated structure (e.g., PFAs, PFTAAs, FEs) were found to be extremely volatile, even if the total molecular size was large. These results have implications with respect to the emissions and environmental fates of PFAS, as well as the exposure of humans and wildlife to these chemicals.

Although the two models tested were both able to predict log *K*_Hxd/air_ well, COSMO*therm* appeared to be more robust across a diverse set of structures. COSMO*therm* may be particularly useful for relative evaluation across various PFAS because the major error source seemed not to be specific to a particular group of PFAS. IFS-QSPR gave accurate predictions when it was used within its applicability domain (i.e., UL ≤ 2). Because IFS-QSPR is available for free on the Internet, we recommend this model as the first choice for predicting missing *K*_Hxd/air_ values for PFAS. If a PFAS of concern is not within the applicability domain of IFS-QSPR, the commercial software COSMO*therm* can be used to reasonably fill this data gap.

In our ongoing study, we are measuring GC retention times for the same set of PFAS on various polar columns, which we expect will provide additional pp-LFER descriptors of specific polar interaction properties. In combination with the present log *K*_Hxd/air_ values, such information will enable accurate prediction of other partition coefficients for PFAS through pp-LFER models.

## Supporting Information Available

Additional descriptions of experimental methods; lists of all chemicals, fitted equations, and measured and predicted data; and additional figures for the experimental data and comparison of molar volume and predicted values (PDF)
