# Supplementary material for: Volatility and Nonspecific van der Waals Interaction Properties of Per- and Polyfluoroalkyl Substances (PFAS): Evaluation Using Hexadecane/Air Partition Coefficients
Source: Environ Sci Technol. 2022 Oct 14;56(22):15737–45. doi: 10.1021/acs.est.2c05804 (PMC9671037; doi:10.1021/acs.est.2c05804)
Supplement: Supplementary file 1 — es2c05804_si_001.pdf [file es2c05804_si_001.pdf]

Supporting Information for "Volatility and nonspecific van der Waals interaction properties of per- and polyfluoroalkyl substances (PFAS): Evaluation using hexadecane/air partition coefficients"

*Jort Hammer & Satoshi Endo**

Health and Environmental Risk Division, National Institute for Environmental Studies (NIES), Onogawa 16-2, 305-8506 Tsukuba, Ibaraki, Japan

*Corresponding author, Satoshi Endo, Phone: ++81-29-850-2695, endo.satoshi@nies.go.jp

(30 pages with 8 tables and 9 figures)

SI-1 GC/MS conditions for VPR-HS methods.

SI-2 Details for GC-RT methods.

SI-3 Possible polar interactions with CP-Squalane and SPB-Octyl columns.

Table S1. List of PFAS used in this study.

Table S2. List of reference chemicals.

Table S3. $K_{Hxd/air}$ values at 25°C determined by the VPR-HS method.

Table S4. Log *k*' values on CP-Squalane (SQ) and SPB-Octyl columns.

Table S5. Calibrated values of fitting coefficients of eq 4 for log *k*'.

Table S6. Calibrated values of fitting coefficients of eq S1 for log *k*'.

Table S7. Log $K_{Hxd/air}$ values at 25°C determined by GC-RT methods.

Table S8. List of experimental and predicted log $K_{Hxd/air}$ values.

Figure S1. GC peak area vs phase ratio (V_{HS}/V_{Hxd}) in the VPR-HS method.

Figure S2. Examples of chromatograms for FEs.

Figure S3. Comparison of log $K_{Hxd/air}$ determined by the VPR-HS method and by the GC retention method using only reference chemicals as calibration data.

Figure S4. Measured log *k*' vs McGowan's molar volume (*V*).

Figure S5. Fitted and experimental log $K_{Hxd/air}$ values for chemicals used for calibration of eq 4.

Figure S6. Measured log $K_{Hxd/air}$ for all PFAS.

Figure S7. Experimental vs COSMO*therm*-predicted log $K_{Hxd/air}$ for reference chemicals and PFAS.

Figure S8. Prediction errors (predicted minus experimental) and uncertainty levels (UL) provided by IFS-QSPR.

Figure S9. Experimental log $K_{Hxd/air}$ values vs predicted values by a **former version** of IFS-QSPR.

SI-1 GC/MS conditions for VPR-HS methods

In the VPR-HS method, the headspace was measured with a 7890A GC/5975C MS (Agilent Technologies) equipped with an MPS2 autosampler (Gerstel). An Rtx-624 column (0.25 mm × 60 m, film thickness 1.4 μm) was used for the analysis. Temperature of the injector and the transfer line was 100 and 240°C, respectively. Flow of the carrier gas (helium) was 1.4 mL/min. Isothermal measurement was conducted, where the oven temperature depended on the chemicals (< 200°C), or an oven temperature program (70°C for 0.5 min, raised to 230°C at a rate of 20°C/min) was applied. Headspace (500 μL) was injected to GC using a 2.5 mL glass syringe in the split injection mode with a split ratio of 4:1 to 50:1. After each measurement, the syringe was flushed with N_2 gas for 1 min. The ion source temperature was 230°C. The MS was operated in the selected ion monitoring (SIM) mode. Two or three major ions (e.g., *m/z* of 69, 77, and 95) were monitored. There was only one major peak in the chromatogram for each of the 16 PFAS that were subjected to the VPR-HS measurement.

SI-2 Details for GC-RT methods

The chemicals were introduced into the column by injection of 250−2500 µL of the headspace above the pure liquid or solid of a test chemical at a speed of 200 µL/s. A split ratio of 10:1 to 250:1 was applied, depending on the chemical's volatility. The injector temperature was the same as the oven temperature. The flow of carrier gas (He) was 1.5 (10-m SQ), 2.0 (75-m SQ), or 1.2 mL/min (SPB-Octyl). For the retention time measurement at 70 and 100°C with SPB-Octyl, acetone solution (1 µL) was injected to GC for chemicals that were not volatile enough for the headspace injection. The injector temperature for the liquid injection was 120°C. The retention time was taken at the highest point in the peak. For each chemical, at least duplicate measurements were performed, which usually showed no difference (<1%). If peak fronting occurred, the loading was reduced by decreasing the injection volume and/or increasing the split ratio. If fronting still occurred, the pure chemical was diluted with hexadecane and the headspace above this hexadecane solution was injected into GC. If peak tailing occurred, the loading was increased and the stability of the retention time was checked. Extensively tailing peaks were not considered, which often occurred with polar chemicals in the chromatograms of the CP-Squalane columns.

SI-3 Possible polar interactions with CP-Squalane and SPB-Octyl columns

Some contributions of polar interactions could exist to the retention of nonpolar columns including CP-Squalane and SPB-Octyl columns because of interactions with, e.g., the wall of the capillary column, impurities in the column coating phase, and the siloxane backbone (for SPB-Octyl). To evaluate the potential influences of polar interactions on the measurement of log $K_{\mathrm{Hxd/air}}$, the following equation was fitted to the data sets:

$$\mathrm{Log}\ K_{\mathrm{Hxd/air}} = m \log k' + vV + aA + sS + c \qquad \text{(S1)}$$

Equation S1 includes the terms with A (solute hydrogen bond acid property) and S (polarity/dipolarity parameter), as compared to eq 4 in the manuscript. The hydrogen bond basicity descriptor (B) was omitted because this is usually negligible in GC retention measurement.[1] For this calculation, the A and S descriptors predicted with the IFS-QSPR[2] implemented in EAS-E Suite[3] (accessed on July 15. 2022) were used for PFAS, because experimental values for A and S were not available. The results of fitting are shown in Table S6. In many cases, the fitting coefficients for A and S (i.e., a and s, respectively) were small and not significantly different from 0. The log $K_{\mathrm{Hxd/air}}$ values for PFAS calculated from eq S1 would be different from those from eq 4 in the main manuscript by 0.02–0.10 log units on average, and 0.07–0.18 log units in the worst case. These deviations are well within the 95% prediction intervals of log $K_{\mathrm{Hxd/air}}$ determined by eq 4 and thus are within the range of method uncertainty. Therefore, polar interactions would have no or only a small influence on the measured $K_{\mathrm{Hxd/air}}$ values.

Table S1. List of PFAS used in this study.

Name	Abbreviation	CAS-RN	Group	Provider	Purity
1H,1H-Heptafluoro-1-butanol	3:1 FTOH	375-01-9	FTOHs	TCI	98
3-(Perfluoropropyl)propanol	3:3 FTOH	679-02-7	FTOHs	Apollo Scientific	95
2-(Perfluorobutyl)ethanol	4:2 FTOH	2043-47-2	FTOHs	TCI	97
5,5,6,6,7,7,8,8,8-Nonafluorooctan-1-ol	4:4 FTOH	3792-02-7	FTOHs	SynQuest	98
2-(Perfluorohexyl)ethanol	6:2 FTOH	647-42-7	FTOHs	TCI	98
1H,1H-Perfluorooctan-1-ol	7:1 FTOH	307-30-2	FTOHs	SynQuest	98
2-(Perfluorooctyl)ethanol	8:2 FTOH	678-39-7	FTOHs	TCI	97
2-(Perfluorodecyl)ethanol	10:2 FTOH	865-86-1	FTOHs	SynQuest	97
1H,1H,2H,2H-Perfluorotetradecan-1-ol	12:2 FTOH	39239-77-5	FTOHs	SynQuest	82
3,3,4,4,5,5,6,6,7,7,7-Undecafluoroheptan-2-ol	5:2s FTOH	914637-05-1	FTOHs	SynQuest	97
3-(Perfluoro-2-butyl)propane-1,2-diol	NFHp-1,2-diol	125070-38-4	Others	SynQuest	98
Pentafluoropropanoic anhydride	PFPrAnhy	356-42-3	Others	Sigma-Aldrich	99
N-Ethyl perfluorohexane sulfonamidoethanol	EtFHxSE	34455-03-3	FASEs	SynQuest	99
N-Ethyl perfluorooctane sulfonamidoethanol	EtFOSE	1691-99-2	FASEs	SynQuest	95(sum of isomers)
N-Methyl perfluorobutane sulfonamidoethanol	MeFBSE	34454-97-2	FASEs	TRC	97
N-Methyl perfluorooctane sulfonamidoethanol	MeFOSE	24448-09-7	FASEs	SynQuest	95(sum of isomers)
Perfluorobutane sulfonamide	PFBSA	30334-69-1	FASAs	SynQuest	97
Perfluorohexane sulfonamide	PFHxSA	41997-13-1	FASAs	SynQuest	95(sum of isomers)
Perfluorooctane sulfonamide	PFOSA	754-91-6	FASAs	SynQuest	85(sum of isomers)
N-Methyl perfluorobutane sulfonamide	MeFBSA	68298-12-4	FASAs	SynQuest	97
N-Methyl perfluorohexane sulfonamide	MeFHxSA	68259-15-4	FASAs	SynQuest	97
N-Methyl perfluorooctane sulfonamide	MeFOSA	31506-32-8	FASAs	TRC	97
N-Ethyl perfluorohexane sulfonamide	EtFHxSA	87988-56-5	FASAs	SynQuest	97
N-Ethyl perfluorooctane sulfonamide	EtFOSA	4151-50-2	FASAs	SynQuest	95

Perfluorobutanesulfonyl fluoride	PFBSF	375-72-4	Others	SynQuest	97
Perfluorobutyl iodide	PFBI	423-39-2	PFIs	TCI	98
Perfluorohexyl iodide	PFHxI	355-43-1	PFIs	SynQuest	98
Perfluoroheptyl iodide	PFHpI	335-58-0	PFIs	SynQuest	97
Perfluorooctyl iodide	PFOI	507-63-1	PFIs	SynQuest	98
Perfluorodecyl iodide	PFDI	423-62-1	PFIs	TCI	98
1,8-Diiodoperfluorooctane	1,8-DIPFO	335-70-6	PFIs	Sigma-Aldrich	98
4:2 Fluorotelomer iodide	4:2 FTI	2043-55-2	FTIs	TCI	99
1H,1H-Tridecafluoro-1-iodoheptane	6:1 FTI	212563-43-4	FTIs	Fujifilm-Wako	97
1H,1H,7H-Perfluoroheptyl iodide	6:1 FTI-7H	376-32-9	FTIs	Fluorochem	98
6:2 Fluorotelomer iodide	6:2 FTI	2043-57-4	FTIs	TCI	97
8:2 Fluorotelomer iodide	8:2 FTI	2043-53-0	FTIs	TCI	98
10:2 Fluorotelomer iodide	10:2 FTI	2043-54-1	FTIs	Sigma-Aldrich	95
1,1,1,2,2,3,3-Heptafluoro-3-[(1,1,1,2,3,3-hexafluoro-3-{[1,1,1,2,3,3-hexafluoro-3-(1,2,2,2-tetrafluoroethoxy)-2-propanyl]oxy}-2-propanyl)oxy]propane	FE-E3	3330-16-3	FEs	SynQuest	99(sum of isomers)
1,1,1,2,4,4,5,7,7,8,10,10,11,13,13,14,14,15,15,15-Eicosafluoro-5,8,11-tris(trifluoromethyl)-3,6,9,12-tetraoxapentadecane	FE-E4	26738-51-2	FEs	SynQuest	97
1,1,1,2,4,4,5,7,7,8,10,10,11,13,13,14,16,16,17,17,18,18,18-Tricosafluoro-5,8,11,14-tetrakis(trifluoromethyl)-3,6,9,12,15-pentaoxaoctadecane	FE-E5	37486-69-4	FEs	TCI	95
Allyl 1H,1H-perfluorooctyl ether	AFOE	812-72-6	Others	SynQuest	97
1-(Heptafluoropropoxy)-1,2,2,2-tetrafluoro-1-iodoethane	FE-E1-I	107432-46-2	Others	Apollo Scientific	97
Allyl perfluoroisopropyl ether	APFIPE	15242-17-8	Others	SynQuest	95
Perfluorotripropyl amine	PFTPrA	338-83-0	PFTAAs	Combi-Blocks	95
Perfluorotributyl amine	PFTBA	311-89-7	PFTAAs	Fujifilm-Wako	65 (MS calibrant)

Perfluoroheptane	PFHp	335-57-9	PFAs	Apollo Scientific	97
Perfluorooctane	PFO	307-34-6	PFAs	Apollo Scientific	99
Perfluorononane	PFN	375-96-2	PFAs	Fujifilm-Wako	98
Perfluorododecane	PFDoD	307-59-5	PFAs	SynQuest	97
1H,8H-Perfluorooctane	1,8-DHPFO	307-99-3	Others	SynQuest	97
1,8-Divinylperfluorooctane	1,8-DVPFO	35192-44-0	Others	SynQuest	95
4-(Perfluorooct-1-yl)styrene	PFOSt	106209-21-6	Others	SynQuest	97
4:2 Fluorotelomer olefin	4:2 FTO	19430-93-4	FTOs	TCI	98
6:2 Fluorotelomer olefin	6:2 FTO	25291-17-2	FTOs	TCI	96
8:2 Fluorotelomer olefin	8:2 FTO	21652-58-4	FTOs	TCI	97
6:2 Fluorotelomer acrylate	6:2 FTAC	17527-29-6	FTACs	TCI	98
8:2 Fluorotelomer acrylate	8:2 FTAC	27905-45-9	FTACs	TCI	97
10:2 Fluorotelomer acrylate	10:2 FTAC	17741-60-5	FTACs	Sigma-Aldrich	96
4:2 Fluorotelomer methacrylate	4:2 FTMAC	1799-84-4	FTMACs	TCI	98
6:2 Fluorotelomer methacrylate	6:2 FTMAC	2144-53-8	FTMACs	TCI	98
8:2 Fluorotelomer methacrylate	8:2 FTMAC	1996-88-9	FTMACs	TCI	98
10:2 Fluorotelomer methacrylate	10:2 FTMAC	2144-54-9	FTMACs	TRC	98
4-(3,3,4,4,5,5,6,6,7,7,8,8,8-Tridecafluorooctyl)benzyl alcohol	6:2 FTBnOH	356055-76-0	Others	Sigma-Aldrich	97
1H,1H,2H,2H-Perfluorodecyl acetate	8:2 FTAce	37858-04-1	Others	TRC	98

Abbreviations used: TCI, Tokyo Chemical Industry; TRC, Toronto Research Chemicals; SynQuest, SynQuest Laboratories; Fujifilm-Wako, FUJIFILM Wako Pure Chemical Corporation

Table S2. List of reference chemicals.

Name	CAS-RN	Provider	Purity	Log $K_{Hxd/air}$	V
Carbon disulphide	75-15-0	Fujifilm-Wako	99	2.370	0.4905
Dichloromethane	75-09-2	Fujifilm-Wako	99.5	2.019	0.4943
Trichloromethane	67-66-3	Fujifilm-Wako	99	2.480	0.6167
Tetrahydrofuran	109-99-9	Fujifilm-Wako	99.5	2.636	0.6223
1,2-Dichloroethane	107-06-2	Fujifilm-Wako	99.5	2.573	0.6352
Dimethylformamide	68-12-2	TCI	99.5	3.173	0.6468
2-Methylpropane	75-28-5	STYLE	In Prince GB-2001 LPG	1.409	0.6722
n-Butane	106-97-8	STYLE	In Prince GB-2001 LPG	1.615	0.6722
Pyridine	110-86-1	Fujifilm-Wako	99.5	3.022	0.6753
2-Butanone	78-93-3	Fujifilm-Wako	99	2.287	0.6879
1-Nitropropane	108-03-2	Fujifilm-Wako	97.0	2.894	0.7055
Benzene	71-43-2	Fujifilm-Wako	99.7	2.786	0.7164
Diethyl ether	60-29-7	Fujifilm-Wako	99.5	2.015	0.7309
Butan-1-ol	71-36-3	Nacalai tesque	99	2.601	0.7309
Ethyl acetate	141-78-6	Fujifilm-Wako	99.7	2.314	0.7466
n-Pentane	109-66-0	STYLE	In Prince GB-2001 LPG	2.162	0.8131
2-Methylbutane	78-78-4	STYLE	In Prince GB-2001 LPG	2.013	0.8131
Resorcinol	108-46-3	Fujifilm-Wako	99	4.829	0.8338
Cyclohexane	110-82-7	Fujifilm-Wako	99.7	2.964	0.8454
Toluene	108-88-3	Fujifilm-Wako	99.7	3.325	0.8573
Cyclohexanone	108-94-1	TCI	99	3.792	0.8611
Pentan-1-ol	71-41-0	Nacalai tesque	98	3.106	0.8718
2-Chlorophenol	95-57-8	Fujifilm-Wako	98.0	4.178	0.8975
4-Chlorophenol	106-48-9	Fujifilm-Wako	98.0	4.775	0.8975
Cyclohexanol	108-93-0	Sigma-Aldrich	99	3.732	0.9041
n-Pentylamine	110-58-7	TCI	98	3.139	0.9129
Methyl phenyl ether	100-66-3	Fujifilm-Wako	99.0	3.890	0.9160
n-Hexane	110-54-3	Fujifilm-Wako	96	2.668	0.9540
1,2-Dichlorobenzene	95-50-1	TCI	99.0	4.518	0.9612
trans-Cyclohexane-1,2-diol	1460-57-7	Fujifilm-Wako	97	4.182	0.9628
Trimethyl phosphate	512-56-1	Fujifilm-Wako	99.0	3.798	0.9707

Benzamide	55-21-0	TCI	99.0	5.277	0.9728
4-Nitroaniline	100-01-6	Fujifilm-Wako	99	6.358	0.9904
n-Butyl acetate	123-86-4	Fujifilm-Wako	99.0	3.409	1.0284
2-Phenylethanol	60-12-8	Fujifilm-Wako	98.0	4.702	1.0569
2,6-Dimethylphenol	576-26-1	TCI	99	4.680	1.0569
1,3-Dinitrobenzene	99-65-0	Sigma-Aldrich	97	5.903	1.0648
Phenyl acetate	122-79-2	TCI	98	4.414	1.0726
1,2,4-Trichlorobenzene	120-82-1	Sigma-Aldrich	99	5.248	1.0836
n-Heptane	142-82-5	Fujifilm-Wako	99.0	3.173	1.0949
1-Nitrohexane	646-14-0	Sigma-Aldrich	98	4.416	1.1282
Octan-2-one	111-13-7	Fujifilm-Wako	98.0	4.257	1.2515
Di-n-butyl ether	142-96-1	Fujifilm-Wako	98.0	3.924	1.2945
Octan-1-ol	111-87-5	TCI	99.5	4.619	1.2945
Methyl cinnamate	103-26-4	Fujifilm-Wako	98.0	5.890	1.3114
n-Nonane	111-84-2	Nacalai tesque	98.0	4.182	1.3767
Triethyl phosphate	78-40-0	Fujifilm-Wako	97.0	4.750	1.3934
Dimethyl phthalate	131-11-3	Fujifilm-Wako	98	6.275	1.4288
n-Decane	124-18-5	Nacalai tesque	99.5	4.686	1.5176
Decan-1-ol	112-30-1	TCI	98	5.610	1.5763
n-Undecane	1120-21-4	Sigma-Aldrich	Alkane standard solution C8-C20	5.191	1.6585
n-Dodecane	112-40-3	Sigma-Aldrich	Alkane standard solution C8-C20	5.696	1.7994
2-Dodecanone	6175-49-1	TCI	98	6.184	1.8151
Tri-n-propyl phosphate	513-08-6	Sigma-Aldrich	99	6.180	1.8161
Dihexyl ether	112-58-3	Sigma-Aldrich	97	5.938	1.8581
Dodecan-1-ol	112-53-8	TCI	99	6.620	1.8581
n-Tridecane	629-50-5	Sigma-Aldrich	Alkane standard solution C8-C20	6.200	1.9403
n-Tetradecane	629-59-4	Sigma-Aldrich	Alkane standard solution C8-C20	6.705	2.0812
Octamethyltrisiloxane (L3)	107-51-7	Sigma-Aldrich	98	3.936	2.0903
Octamethylcyclotetrasiloxane (D4)	556-67-2	Fujifilm-Wako	95	4.473	2.3448
Decamethylcyclo pentasiloxane (D5)	541-02-6	TCI	99	5.242	2.9310

Abbreviations used: TCI, Tokyo Chemical Industry; Fujifilm-Wako, FUJIFILM Wako Pure Chemical Corporation.

Table S3. $K_{Hxd/air}$ values at 25°C determined by the VPR-HS method.

	$K_{Hxd/air}$	95% CI (low)	95% CI (high)	log $K_{Hxd/air}$
3:1 FTOH	35	28	42	1.54
3:3 FTOH	490	448	544	2.69
4:2 FTOH	232	215	251	2.37
5:2s FTOH	143	123	171	2.16
PFPrAnhy	41	34	49	1.62
PFBI	85	78	94	1.93
FE-E3	60	58	62	1.78
FE-E4	218	206	232	2.34
FE-E5	452	355	600	2.66
FE-E1-I	93	87	100	1.97
APFIPE	54	52	57	1.74
PFO	21	17	26	1.33
PFN	36	31	42	1.56
4:2 FTO	28	24	32	1.44
6:2 FTO	113	93	140	2.05
8:2 FTO	264	243	289	2.42

Table S4. Log *k*' values on CP-Squalane (SQ) and SPB-Octyl columns.

	SQ 75 m, 30°C	SQ 10 m, 30°C	SPB-Octyl, 30°C	SPB-Octyl, 70°C	SPB-Octyl, 100°C
Carbon disulphide			-0.26		
Dichloromethane	-0.54		-0.56		
Trichloromethane	-0.10	-0.01	-0.12	-0.66	
Tetrahydrofuran			-0.05	-0.61	
1,2-Dichloroethane			-0.02		
Dimethylformamide			0.42	-0.23	
2-Methylpropane	-1.13				
n-Butane	-0.95				
Pyridine			0.44	-0.22	
2-Butanone			-0.30		
1-Nitropropane			0.22	-0.39	
Benzene	0.18	0.25	0.15		
Diethyl ether			-0.60		
Butan-1-ol			-0.02	-0.61	
Ethyl acetate			-0.23		
n-Pentane	-0.46		-0.54		
2-Methylbutane	-0.58				
Resorcinol					0.61
Cyclohexane	0.30	0.37	0.23	-0.35	
Toluene			0.64	-0.03	
Cyclohexanone			0.98	0.26	-0.18
Pentan-1-ol			0.46	-0.24	
2-Chlorophenol				0.64	0.16
4-Chlorophenol				1.12	0.54
Cyclohexanol			0.99	0.25	-0.19
n-Pentylamine				-0.19	-0.49
Methyl phenyl ether		1.31	1.20	0.42	-0.04
n-Hexane	0.03	0.11	-0.05	-0.64	
1,2-Dichlorobenzene				0.86	0.36
trans-Cyclohexane-1,2-diol					0.22
Trimethyl phosphate				0.20	0.05
Benzamide					0.92
4-Nitroaniline					1.54

n-Butyl acetate			0.68	-0.05	-0.27
2-Phenylethanol				0.96	0.43
2,6-Dimethylphenol				0.99	0.46
1,3-Dinitrobenzene					1.28
Phenyl acetate				0.78	0.26
1,2,4-Trichlorobenzene					0.81
n-Heptane	0.51	0.56	0.40		
1-Nitrohexane				0.70	0.19
Octan-2-one				0.60	0.09
Di-n-butyl ether		1.34	1.14	0.33	-0.16
Octan-1-ol					0.31
Methyl cinnamate					1.23
n-Nonane		1.48		0.46	-0.04
Triethyl phosphate					0.34
Dimethyl phthalate					1.29
n-Decane		1.95		0.81	0.27
Decan-1-ol					0.92
n-Undecane					0.57
n-Dodecane					0.87
2-Dodecanone					1.29
Tri-n-propyl phosphate					1.14
Dihexyl ether					1.03
Dodecan-1-ol					1.51
n-Tridecane					1.16
n-Tetradecane					1.46
Octamethyltrisiloxane (L3)			1.04	0.19	-0.31
Octamethylcyclotetrasiloxane (D4)			1.50	0.54	-0.01
Decamethylcyclo pentasiloxane (D5)					0.45
3:1 FTOH			-0.78		
3:3 FTOH			0.20	-0.53	
4:2 FTOH			-0.08		
4:4 FTOH				0.02	-0.46
6:2 FTOH			0.41	-0.40	
7:1 FTOH			0.24		
8:2 FTOH				-0.04	-0.56
10:2 FTOH				0.31	-0.27
12:2 FTOH				0.67	0.02

5:2s FTOH			-0.04		
NFHp-1,2-diol				0.02	-0.46
PFPrAnhy	-1.51				
EtFHxSE					0.80
EtFOSE					1.07
MeFBSE					0.33
MeFOSE					0.88
PFBSA				0.26	-0.34
PFHxSA				0.63	-0.05
PFOSA					0.23
MeFBSA				0.14	-0.39
MeFHxSA				0.49	-0.10
MeFOSA				0.84	0.18
EtFHxSA				0.66	0.05
EtFOSA				1.01	0.33
PFBSF	-1.23				
PFBI	-0.61	-0.54	-0.69		
PFHxI	-0.07	-0.03	-0.18		
PFHpI	0.19	0.21	0.06	-0.61	
PFOI	0.46	0.48	0.30	-0.45	
PFDI	0.99	0.99	0.78	-0.09	-0.59
1,8-DIPFO					0.41
4:2 FTI	0.72	0.73	0.58	-0.16	-0.59
6:1 FTI	0.80	0.78	0.62		
6:1 FTI-7H		1.27		0.26	-0.24
6:2 FTI		1.26	1.05	0.20	-0.29
8:2 FTI		1.77		0.54	-0.02
10:2 FTI				0.89	0.27
FE-E3	-0.52	-0.50	-0.72		
FE-E4	0.18	-0.02	-0.03		
FE-E5	0.92		0.67		
AFOE	1.00	0.96	0.78		
FE-E1-I	-0.64		-0.70		
APFIPE	-0.82		-0.89		
PFTPrA	-0.97		-1.14		
PFTBA	-0.22		-0.43		
PFHp	-1.42				

PFO	-1.15				
PFN	-0.95				
PFDoD	-0.12	-0.26	-0.34		
1,8-DHPFO	-0.32	-0.38	-0.44		
1,8-DVPFO		1.26	1.11	0.20	-0.33
PFOSt				1.38	0.69
4:2 FTO	-1.09		-1.08		
6:2 FTO	-0.56	-0.41	-0.65		
8:2 FTO	-0.03	0.03	-0.16		
6:2 FTAC				0.34	-0.20
8:2 FTAC				0.69	0.08
10:2 FTAC				1.04	0.36
4:2 FTMAC				0.31	-0.22
6:2 FTMAC				0.66	0.06
8:2 FTMAC				1.01	0.34
10:2 FTMAC				1.35	0.62
6:2 FTBnOH					1.38
8:2 FTAce				0.40	-0.18
(Individual FE isomers)					
FE-E3a	-0.52	-0.50	-0.72		
FE-E3b	-0.51				
FE-E4a	0.15	-0.02	-0.06		
FE-E4b	0.18		-0.03		
FE-E4c	0.19		-0.02		
FE-E5a	0.88		0.63		
FE-E5b	0.89		0.65		
FE-E5c	0.91		0.66		
FE-E5d	0.92		0.67		
FE-E5e	0.93		0.68		
FE-E5f	0.93		0.69		
FE-E5g	0.95		0.70		

Table S5. Calibrated values of fitting coefficients of eq 4 for log *k*'.

	m	*v*	*c*	R^2	SD	*n*
CP-Squalane 75 m, 30°C (ref)	1.040	0.134	2.511	0.9995	0.015	10
	(0.011)	(0.035)	(0.029)			
CP-Squalane 75 m, 30°C (ref + PFAS)	1.058	-0.050	2.658	0.9944	0.044	18
	(0.021)	(0.020)	(0.026)			
CP-Squalane 10 m, 30°C (ref)	1.023	0.181	2.393	0.9983	0.037	9
	(0.037)	(0.085)	(0.065)			
CP-Squalane 10 m, 30°C (ref + PFAS)	1.104	-0.050	2.565	0.9970	0.054	12
	(0.020)	(0.037)	(0.048)			
SPB-Octyl, 30°C (ref)	1.053	0.149	2.541	0.9946	0.051	26
	(0.023)	(0.032)	(0.027)			
SPB-Octyl, 30°C (ref + PFAS)	1.112	0.057	2.586	0.9861	0.090	35
	(0.023)	(0.034)	(0.038)			
SPB-Octyl, 70°C (ref)	1.249	0.268	3.185	0.9954	0.052	29
	(0.019)	(0.027)	(0.028)			
SPB-Octyl, 70°C (ref + PFAS)	1.269	0.247	3.197	0.9939	0.060	30
	(0.021)	(0.030)	(0.032)			
SPB-Octyl, 100°C (ref)	1.540	0.396	3.585	0.991	0.093	39
	(0.027)	(0.032)	(0.044)			

Values in parentheses are the standard errors. (ref), only reference chemicals were used for calibration. (ref + PFAS), reference and selected PFAS were used for calibration.

Table S6. Calibrated values of fitting coefficients of eq S1 for log *k*'.

	m	*v*	*a*	*s*	*c*	R^2	SD	*n*
CP-Squalane 75 m, 30°C (ref)	1.094	-0.098	-0.069	-0.126	2.728	0.9998	0.011	10
	(0.022)	(0.091)	(0.104)	(0.051)	(0.084)			
CP-Squalane 75 m, 30°C (ref + PFAS)	1.062	-0.068	-0.234	-0.069	2.694	0.9957	0.041	18
	(0.020)	(0.021)	(0.323)	(0.064)	(0.030)			
CP-Squalane 10 m, 30°C (ref)	1.184	-0.304	-0.135	-0.275	2.831	0.9996	0.022	9
	(0.051)	(0.147)	(0.185)	(0.080)	(0.130)			
CP-Squalane 10 m, 30°C (ref + PFAS)	1.099	-0.118	-0.330	-0.154	2.685	0.9987	0.039	12
	(0.015)	(0.035)	(0.313)	(0.055)	(0.052)			
SPB-Octyl, 30°C (ref)	1.073	0.108	-0.113	-0.041	2.597	0.9951	0.051	26
	(0.030)	(0.050)	(0.091)	(0.045)	(0.057)			
SPB-Octyl, 30°C (ref + PFAS)	1.126	0.007	-0.422	-0.079	2.696	0.9917	0.072	35
	(0.021)	(0.038)	(0.094)	(0.053)	(0.057)			
SPB-Octyl, 70°C (ref)	1.253	0.269	-0.105	0.024	3.180	0.9961	0.050	29
	(0.026)	(0.041)	(0.059)	(0.035)	(0.060)			
SPB-Octyl, 70°C (ref + PFAS)	1.278	0.236	-0.162	0.013	3.218	0.9954	0.054	30
	(0.026)	(0.043)	(0.059)	(0.039)	(0.063)			
SPB-Octyl, 100°C (ref)	1.588	0.299	-0.062	-0.096	3.764	0.9923	0.089	39
	(0.034)	(0.053)	(0.047)	(0.048)	(0.091)			

Values in parentheses are the standard errors. (ref), only reference chemicals were used for calibration. (ref + PFAS), reference and selected PFAS were used for calibration.

Table S7. Log $K_{Hxd/air}$ values at 25°C determined by GC-RT methods.

	Squalane 75 m, 30°C			Squalane 10 m, 30°C			SPB-Octyl, 30°C			SPB-Octyl, 70°C			SPB-Octyl, 100°C		
	Value	95% PI (low)	95% PI (high)	Value	95% PI (low)	95% PI (high)	Value	95% PI (low)	95% PI (high)	Value	95% PI (low)	95% PI (high)	Value	95% PI (low)	95% PI (high)
3:1 FTOH							1.77	1.58	1.96						
3:3 FTOH							2.88	2.69	3.07	2.85	2.71	2.98			
4:2 FTOH							2.57	2.38	2.76						
4:4 FTOH										3.63	3.50	3.76	3.52	3.32	3.72
6:2 FTOH							3.14	2.95	3.34	3.13	3.00	3.27			
7:1 FTOH							2.96	2.77	3.16						
8:2 FTOH										3.69	3.55	3.84	3.60	3.38	3.81
10:2 FTOH										4.25	4.09	4.41	4.22	4.00	4.44
12:2 FTOH										4.80	4.63	4.98	4.83	4.61	5.06
5:2s FTOH							2.63	2.44	2.82						
NFHp-1,2-diol										3.60	3.47	3.73	3.48	3.28	3.68
PFPrAnhy	0.99	0.88	1.09												
EtFHxSE													5.78	5.58	5.98
EtFOSE													6.37	6.16	6.58
MeFBSE													4.84	4.64	5.03
MeFOSE													6.02	5.81	6.23
PFBSA										3.87	3.75	4.00	3.61	3.41	3.80
PFHxSA										4.44	4.31	4.58	4.23	4.03	4.42
PFOSA													4.84	4.64	5.04
MeFBSA										3.75	3.63	3.88	3.59	3.39	3.79
MeFHxSA										4.30	4.17	4.44	4.21	4.01	4.41
MeFOSA										4.86	4.71	5.01	4.82	4.61	5.02
EtFHxSA										4.55	4.41	4.69	4.49	4.29	4.69
EtFOSA										5.10	4.95	5.26	5.10	4.89	5.31
PFBSF	1.29	1.19	1.39												
PFBI	1.95	1.86	2.05	1.90	1.77	2.04	1.89	1.70	2.08						
PFHxI	2.50	2.40	2.60	2.44	2.31	2.58	2.48	2.29	2.68						
PFHpI	2.76	2.66	2.87	2.69	2.55	2.84	2.76	2.57	2.96	2.89	2.75	3.04			
PFOI	3.03	2.92	3.15	2.99	2.84	3.14	3.04	2.84	3.25	3.15	3.00	3.30			
PFDI	3.57	3.44	3.71	3.53	3.36	3.70	3.60	3.39	3.82	3.71	3.55	3.87	3.69	3.47	3.91
1,8-DIPFO													5.15	4.95	5.36
4:2 FTI	3.34	3.23	3.46	3.30	3.17	3.43	3.32	3.13	3.51	3.37	3.24	3.50	3.29	3.09	3.50
6:1 FTI	3.41	3.29	3.53	3.34	3.20	3.48	3.38	3.19	3.58						
6:1 FTI-7H				3.87	3.73	4.01				3.97	3.84	4.11	3.93	3.73	4.13
6:2 FTI				3.85	3.71	4.00	3.87	3.67	4.07	3.94	3.80	4.07	3.92	3.72	4.12
8:2 FTI				4.40	4.23	4.57				4.48	4.34	4.63	4.51	4.31	4.72
10:2 FTI										5.04	4.87	5.20	5.12	4.91	5.34
FE-E3	1.98	1.86	2.09	1.88	1.69	2.06	1.93	1.71	2.16						
FE-E4	2.68	2.53	2.82	2.37	2.14	2.60	2.75	2.50	3.00						
FE-E5	3.42	3.23	3.60				3.56	3.28	3.85						
AFOE	3.61	3.47	3.74	3.52	3.36	3.67	3.58	3.37	3.78						
FE-E1-I	1.91	1.81	2.01				1.90	1.70	2.09						
APFIPE	1.73	1.63	1.83				1.66	1.47	1.86						

PFTPrA	1.52	1.41	1.63				1.45	1.23	1.67						
PFTBA	2.28	2.15	2.40				2.27	2.04	2.50						
PFHp	1.07	0.97	1.18												
PFO	1.35	1.24	1.45												
PFN	1.55	1.44	1.65												
PFDoD	2.39	2.27	2.52	2.14	1.95	2.32	2.37	2.14	2.59						
1,8-DHPFO	2.23	2.13	2.33	2.06	1.91	2.20	2.20	2.00	2.40						
1,8-DVPFO				3.84	3.68	4.01	3.95	3.74	4.16	4.03	3.88	4.17	4.00	3.79	4.21
PFOSt										5.62	5.46	5.78	5.73	5.52	5.94
4:2 FTO	1.44	1.34	1.54				1.45	1.26	1.65						
6:2 FTO	1.99	1.89	2.08	2.02	1.88	2.16	1.96	1.76	2.16						
8:2 FTO	2.52	2.41	2.63	2.50	2.34	2.65	2.53	2.32	2.73						
6:2 FTAC										4.17	4.03	4.32	4.13	3.93	4.34
8:2 FTAC										4.72	4.57	4.88	4.74	4.53	4.95
10:2 FTAC										5.27	5.10	5.44	5.34	5.12	5.56
4:2 FTMAC										4.06	3.93	4.20	3.99	3.79	4.19
6:2 FTMAC										4.61	4.46	4.75	4.60	4.40	4.80
8:2 FTMAC										5.15	4.99	5.31	5.21	4.99	5.42
10:2 FTMAC										5.70	5.52	5.87	5.80	5.58	6.03
6:2 FTBnOH													6.72	6.51	6.92
8:2 FTAce										4.32	4.17	4.48	4.30	4.08	4.51

Table S8. List of experimental and predicted log $K_{Hxd/air}$ values.

Compound	Experimental	Prediction by COSMOtherm	Prediction by IFS-QSAR (EAS-S)	Prediction by IFS-QSAR (UFZ)	UL for IFS-QSAR (EAS-S)
3:1 FTOH	1.54	2.04	1.77	1.62	1
3:3 FTOH	2.69	3.14	2.62	2.57	1
4:2 FTOH	2.37	3.00	2.40	2.39	0
4:4 FTOH	3.57	4.03	3.39	3.36	0
6:2 FTOH	3.14	3.67	2.96	3.23	0
7:1 FTOH	2.96	3.42	2.89	3.18	0
8:2 FTOH	3.64	4.30	3.52	4.08	0
12:2 FTOH	4.82	5.58	4.64	5.76	1
5:2s FTOH	2.16	3.15	2.72	3.30	0
NFHp-1,2-diol	3.54	3.96	3.68	3.54	0
PFPrAnhy	1.62	1.84	2.43	2.16	1
EtFHxSE	5.78	6.70	6.59	5.92	1
EtFOSE	6.37	7.35	7.15	6.76	1
MeFBSE	4.84	5.63	5.69	4.76	1
MeFOSE	6.02	6.95	6.81	6.45	1
PFBSA	3.74	4.33	3.68	2.88	0
PFHxSA	4.34	5.00	4.24	3.72	0
PFOSA	4.84	5.71	4.80	4.57	0
MeFBSA	3.67	4.53	4.06	3.23	0
MeFHxSA	4.26	5.20	4.62	4.07	0
MeFOSA	4.84	5.84	5.18	4.92	1
EtFHxSA	4.52	5.59	4.96	4.55	0
EtFOSA	5.10	6.22	5.52	5.39	0
PFBSF	1.29	2.48	2.72	2.74	0
PFBI	1.93	2.49	2.68	2.24	0
PFHxI	2.48	3.17	3.24	3.08	0
PFHpI	2.78	3.51	3.52	3.50	1
PFOI	3.05	3.83	3.80	3.93	1
PFDI	3.62	4.49	4.36	4.77	1
1,8-DIPFO	5.15	5.64	6.01	5.75	2
4:2 FTI	3.32	4.04	3.52	3.29	0
6:1 FTI	3.38	4.08	3.74	3.66	0
6:1 FTI-7H	3.93	4.53	4.01	4.47	1

6:2 FTI	3.90	4.71	4.08	4.14	0
8:2 FTI	4.47	5.30	4.64	4.98	1
10:2 FTI	5.08	5.91	5.20	5.82	1
FE-E3	1.78	2.67	3.01	1.59	1
FE-E4	2.34	3.42	3.91	2.07	2
FE-E5	2.66	3.58	4.81	2.54	2
AFOE	3.57	4.28	3.76	3.93	1
FE-E1-I	1.97	2.46	3.02	2.23	0
APFIPE	1.74	2.21	1.82	1.79	0
PFTPrA	1.48	2.57	2.10	1.43	2
PFTBA	2.27	3.45	2.94	2.31	1
PFHp	1.07	1.67	1.31	1.68	1
PFO	1.33	2.01	1.59	2.10	1
PFN	1.56	2.39	1.87	2.52	2
PFDoD	2.30	3.30	2.71	3.79	2
1,8-DHPFO	2.16	2.95	2.13	3.71	1
1,8-DVPFO	3.95	4.60	3.22	4.00	2
PFOSt	5.67	6.25	5.93	6.11	1
4:2 FTO	1.44	1.95	1.29	1.36	0
6:2 FTO	2.05	2.62	1.85	2.21	0
8:2 FTO	2.42	3.32	2.41	3.05	1
10:2 FTO	3.33	3.94	2.97	3.89	1
6:2 FTAC	4.15	4.75	4.19	4.31	0
8:2 FTAC	4.73	5.04	4.75	5.15	0
10:2 FTAC	5.30	5.76	5.31	6.00	1
4:2 FTMAC	4.03	4.42	4.10	4.00	0
6:2 FTMAC	4.60	4.96	4.66	4.85	0
8:2 FTMAC	5.18	5.52	5.22	5.69	0
10:2 FTMAC	5.75	5.95	5.78	6.53	1
6:2 FTBnOH	6.72	6.90	6.62	6.66	0
8:2 FTAce	4.31	5.09	4.35	4.84	1

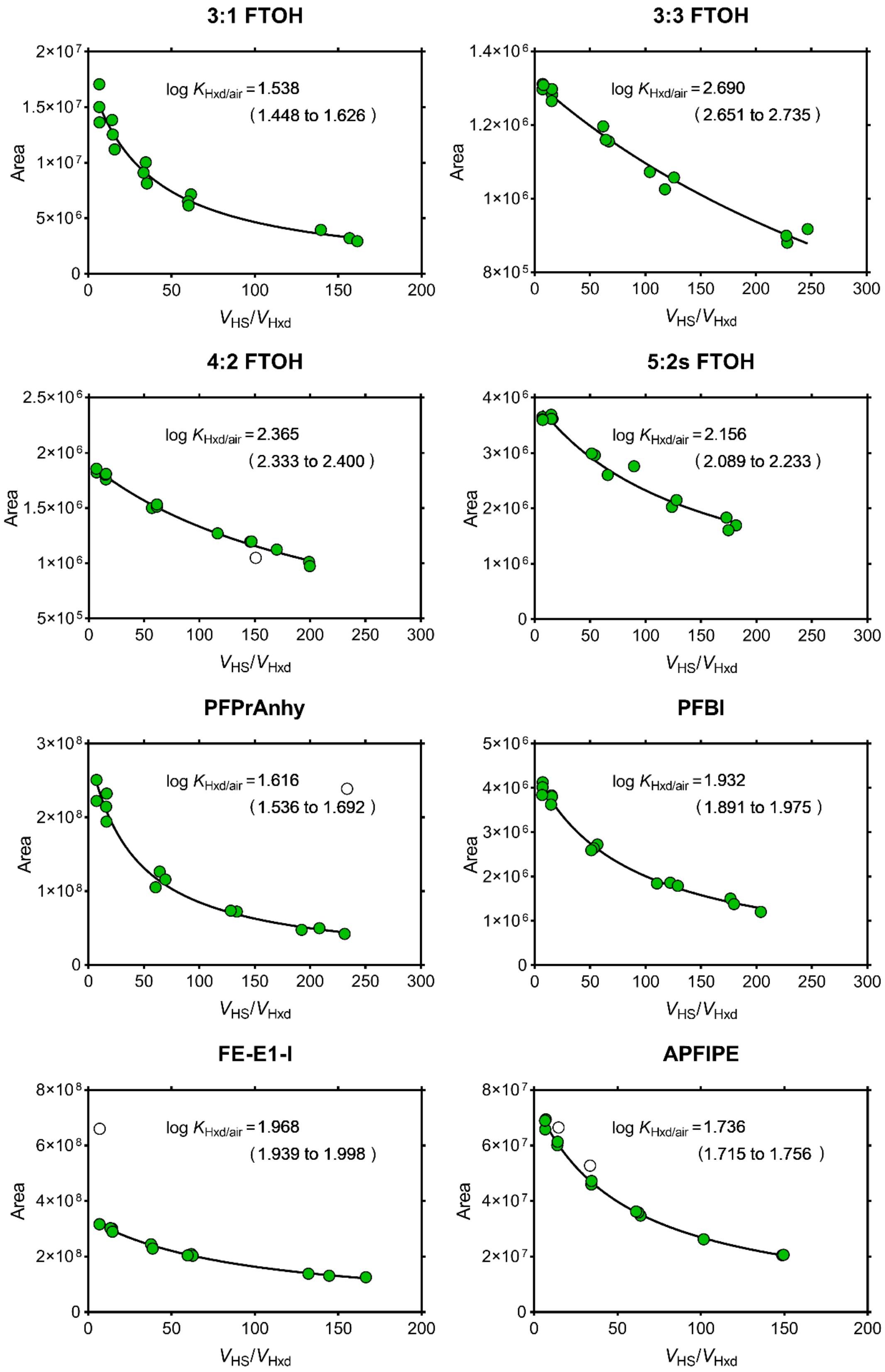

Figure S1. GC peak area vs phase ratio (V_{HS}/V_{Hxd}) in the VPR-HS method. Open symbols indicate statistical outliers (removed from model fitting). The line indicates the result of fitting eq 1.

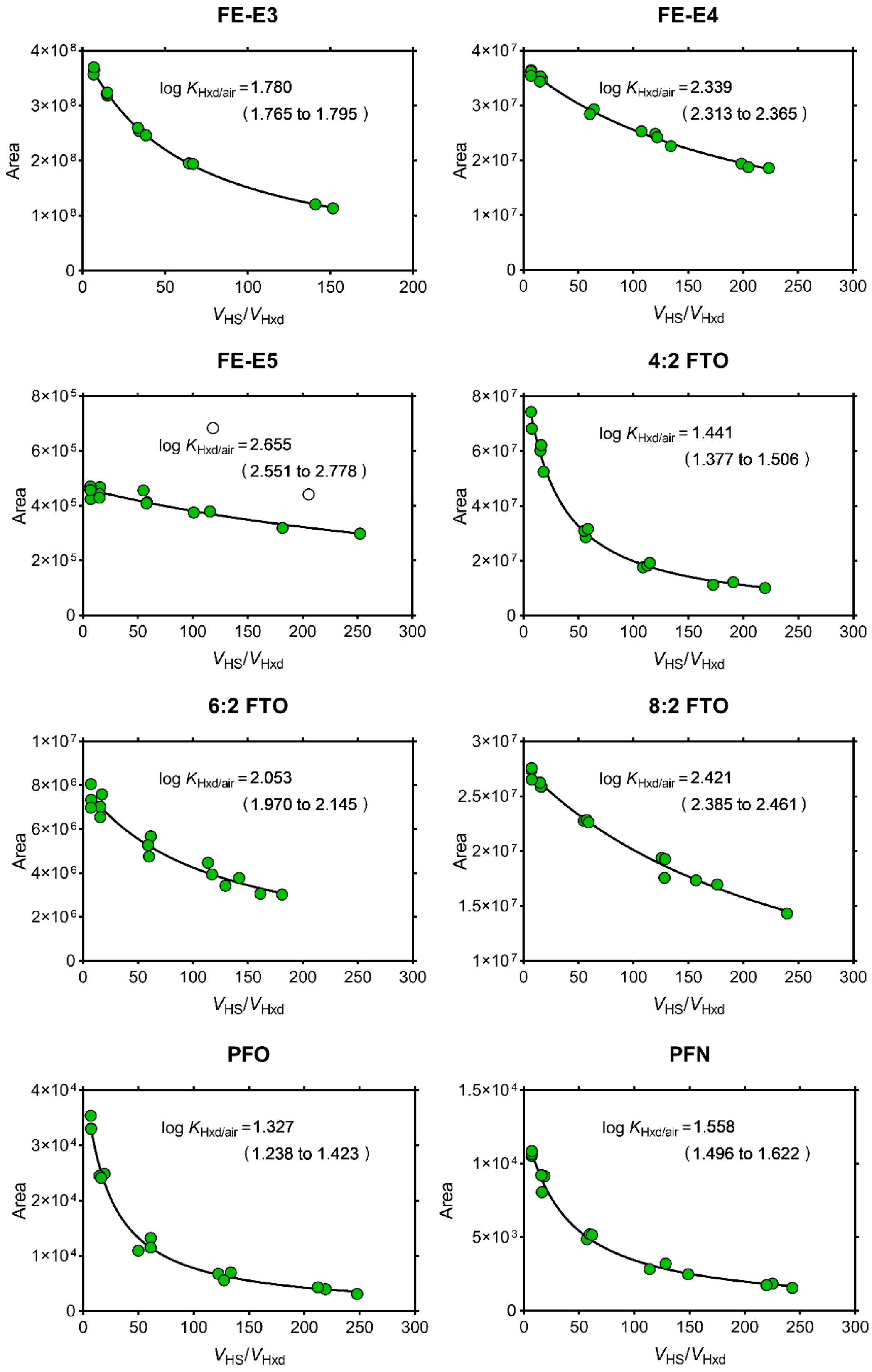

Figure S1 (continued from the previous page)

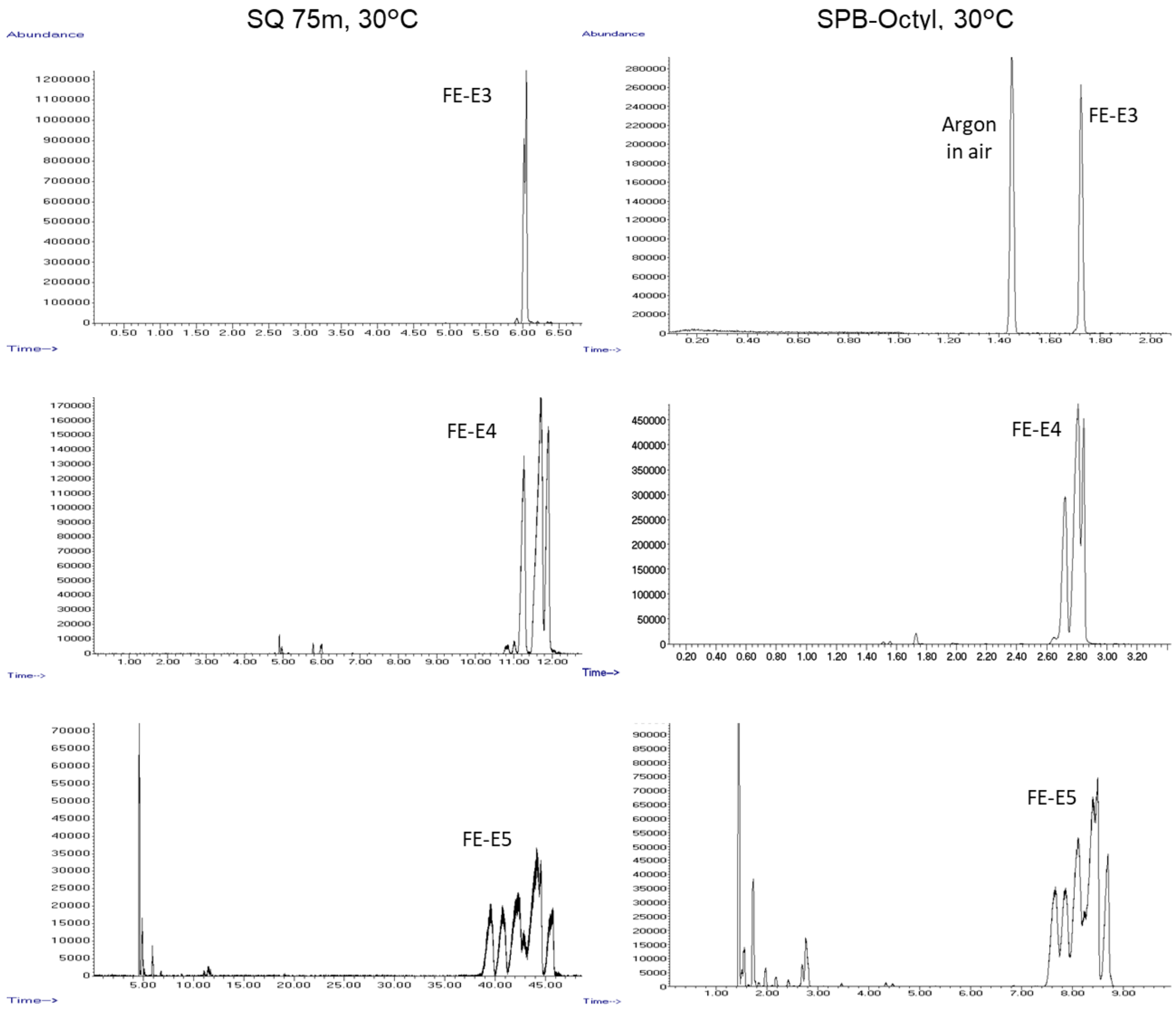

Figure S2. Examples of chromatograms for FEs.

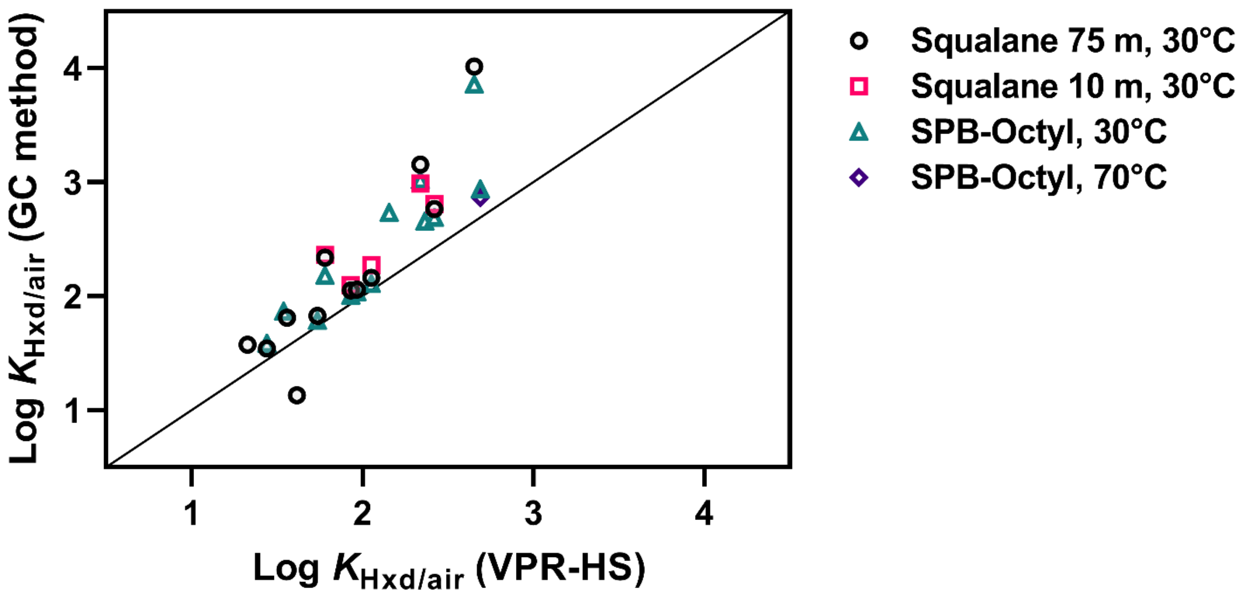

Figure S3. Comparison of log $K_{Hxd/air}$ determined by the VPR-HS method and by the GC retention method using only reference chemicals as calibration data. The line shows the 1:1 relationship.

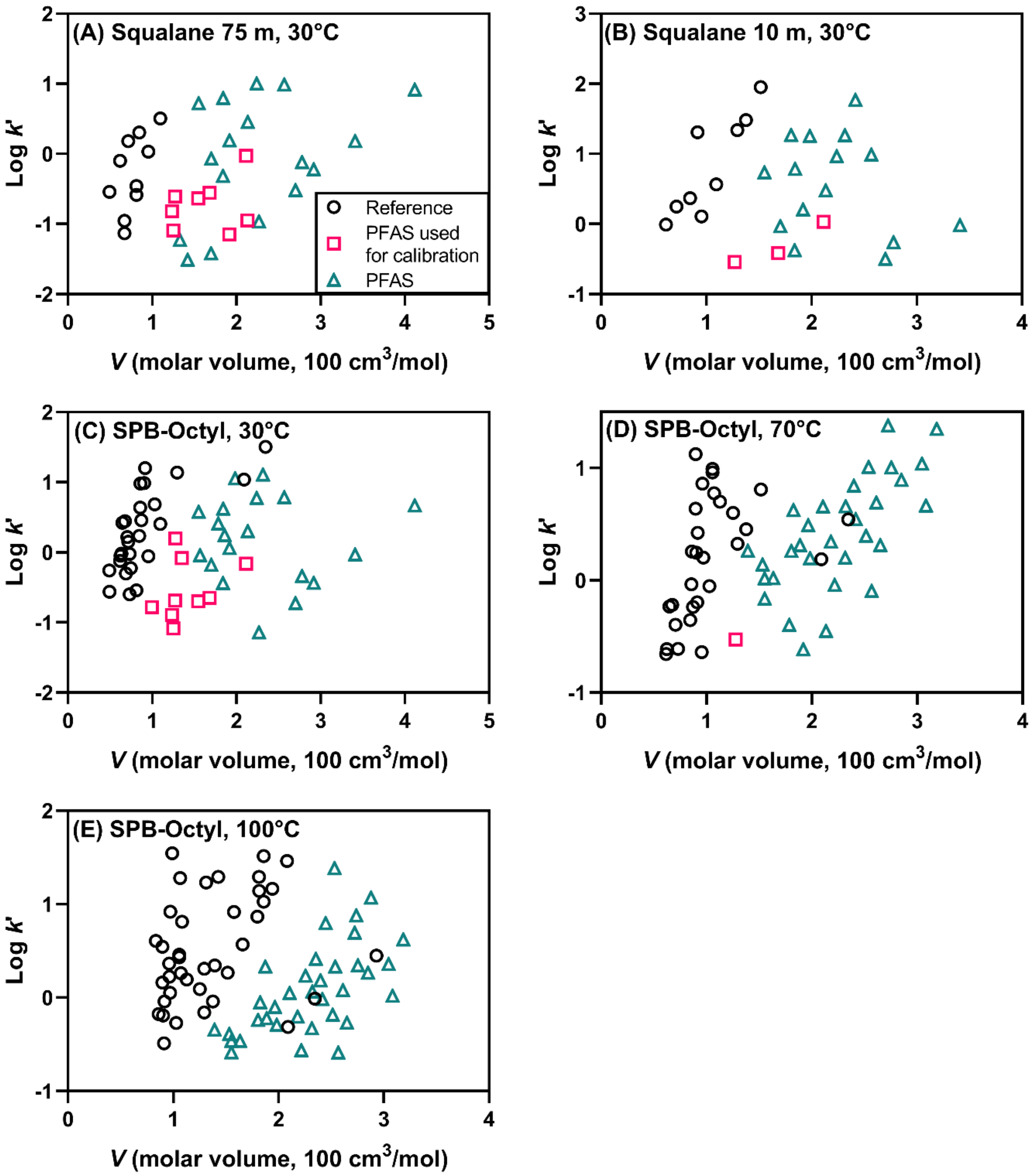

Figure S4. Measured log *k*’ vs McGowan’s molar volume (*V*).

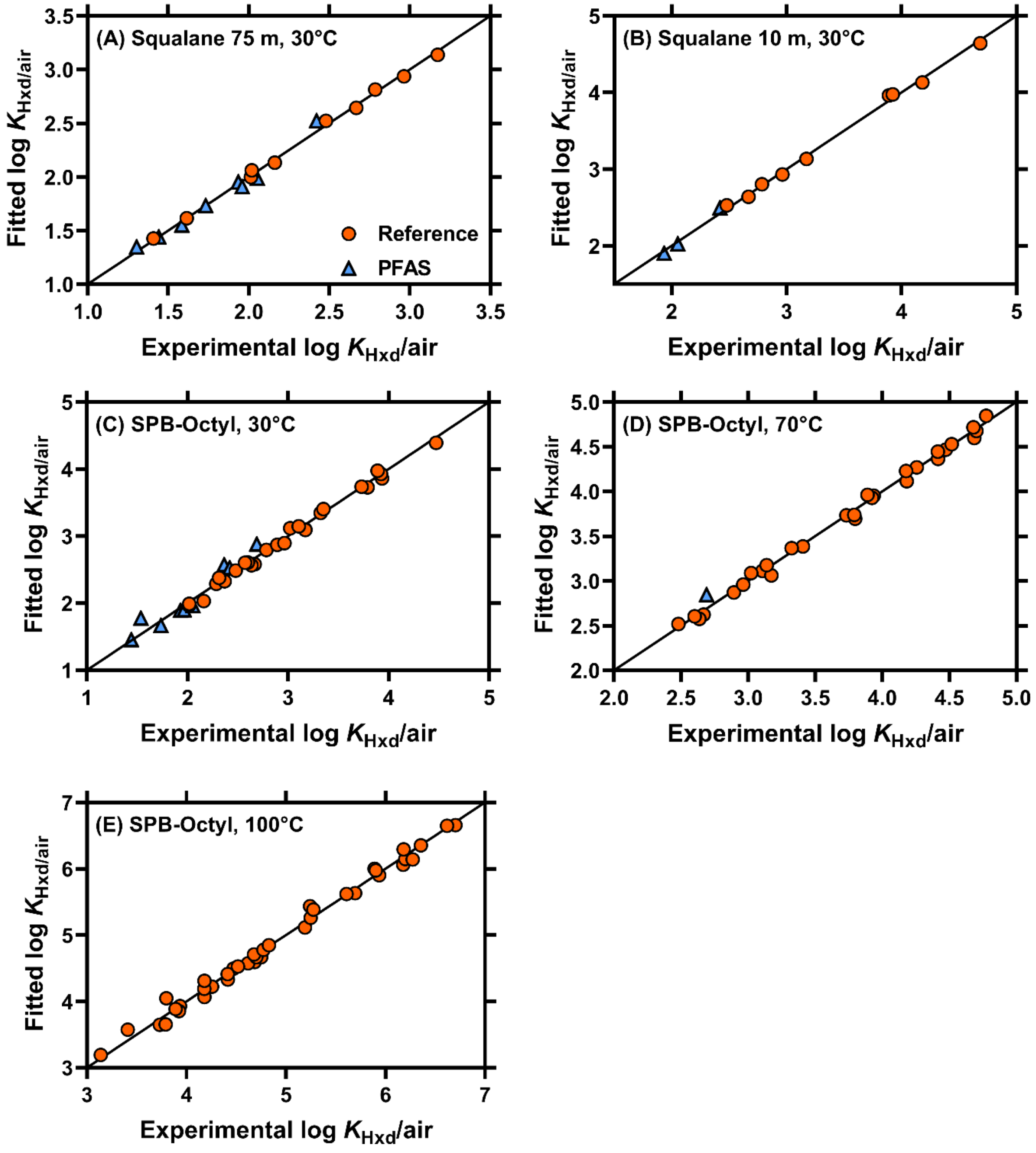

Figure S5. Fitted and experimental log $K_{Hxd/air}$ values for chemicals used for calibration of eq 4. The line shows the 1:1 relationship.

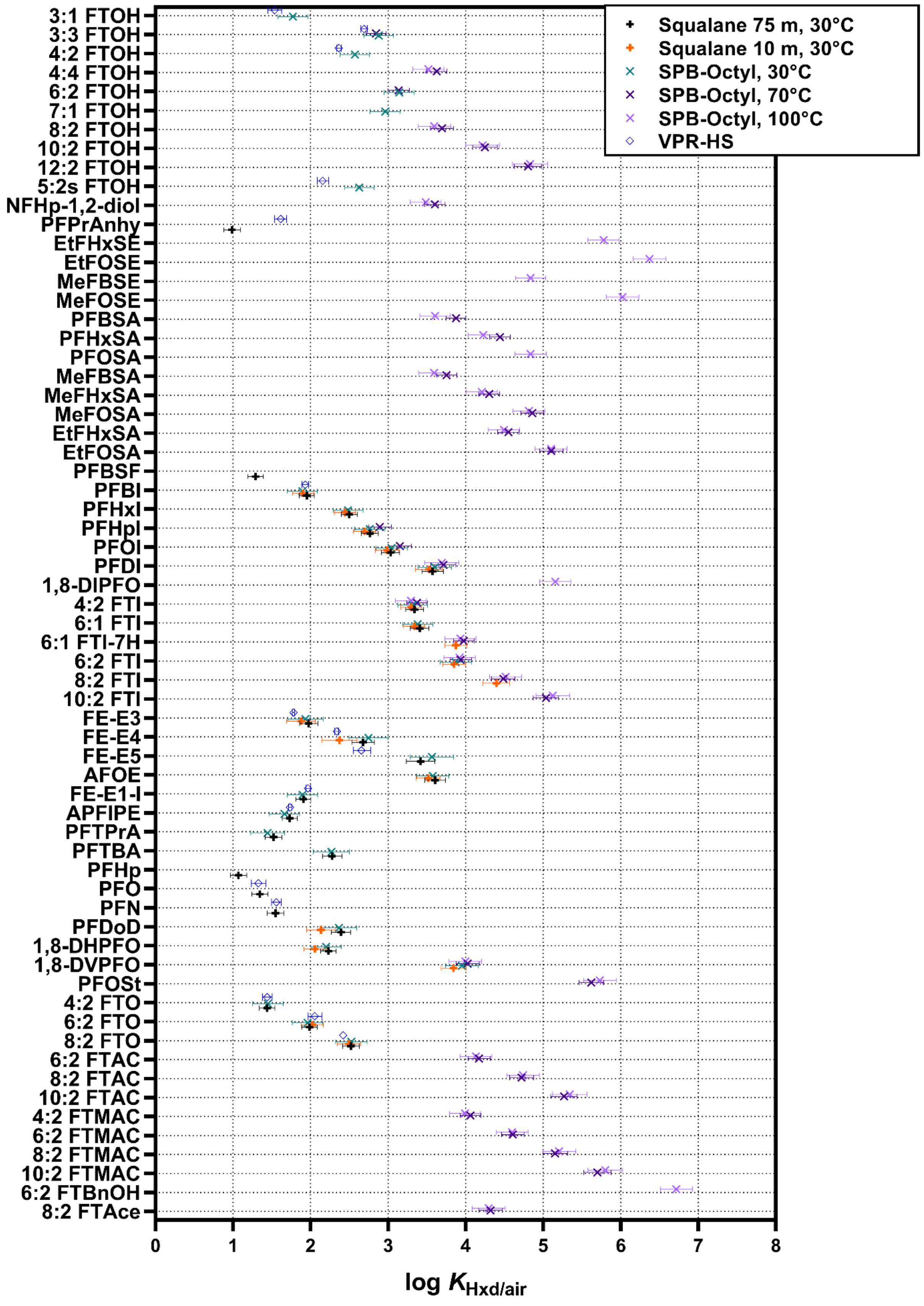

Figure S6. Measured log $K_{\mathrm{Hxd/air}}$ for all PFAS. Error bars indicate the 95% prediction intervals.

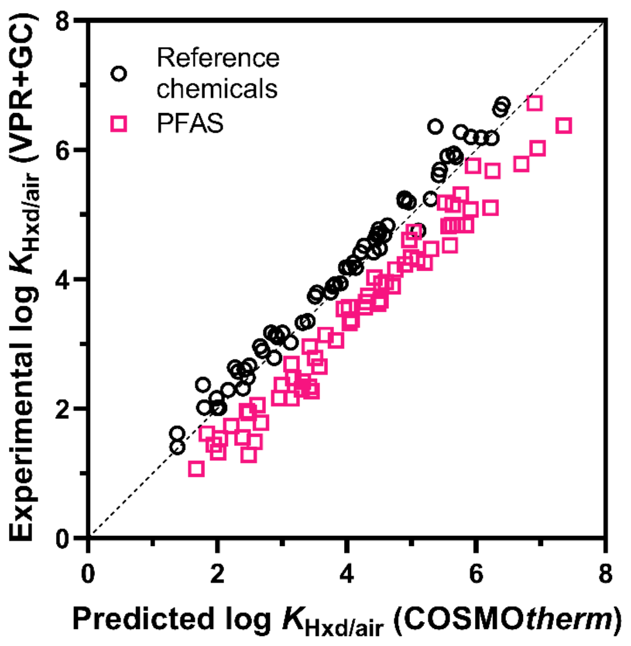

Figure S7. Experimental vs COSMO*therm*-predicted log $K_{Hxd/air}$ for reference chemicals and PFAS. The line indicates the 1:1 line.

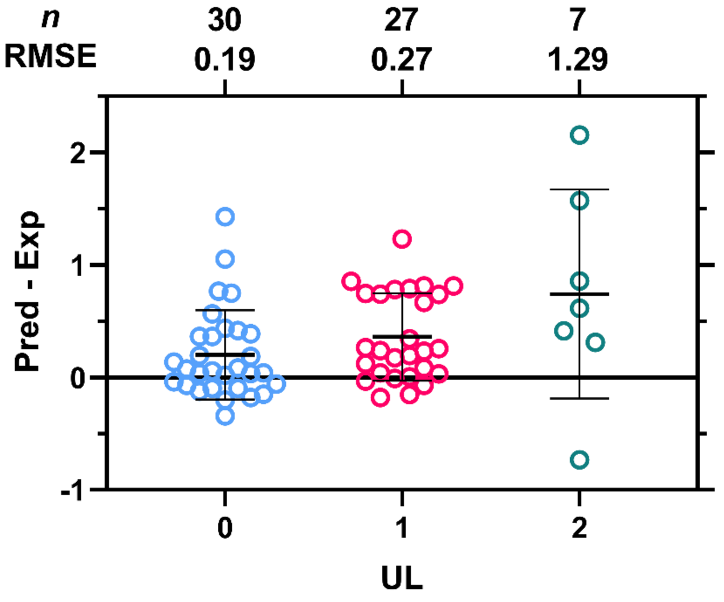

Figure S8. Prediction errors (predicted minus experimental) and uncertainty levels (UL) provided by IFS-QSPR. Lines indicate the mean and standard deviation.

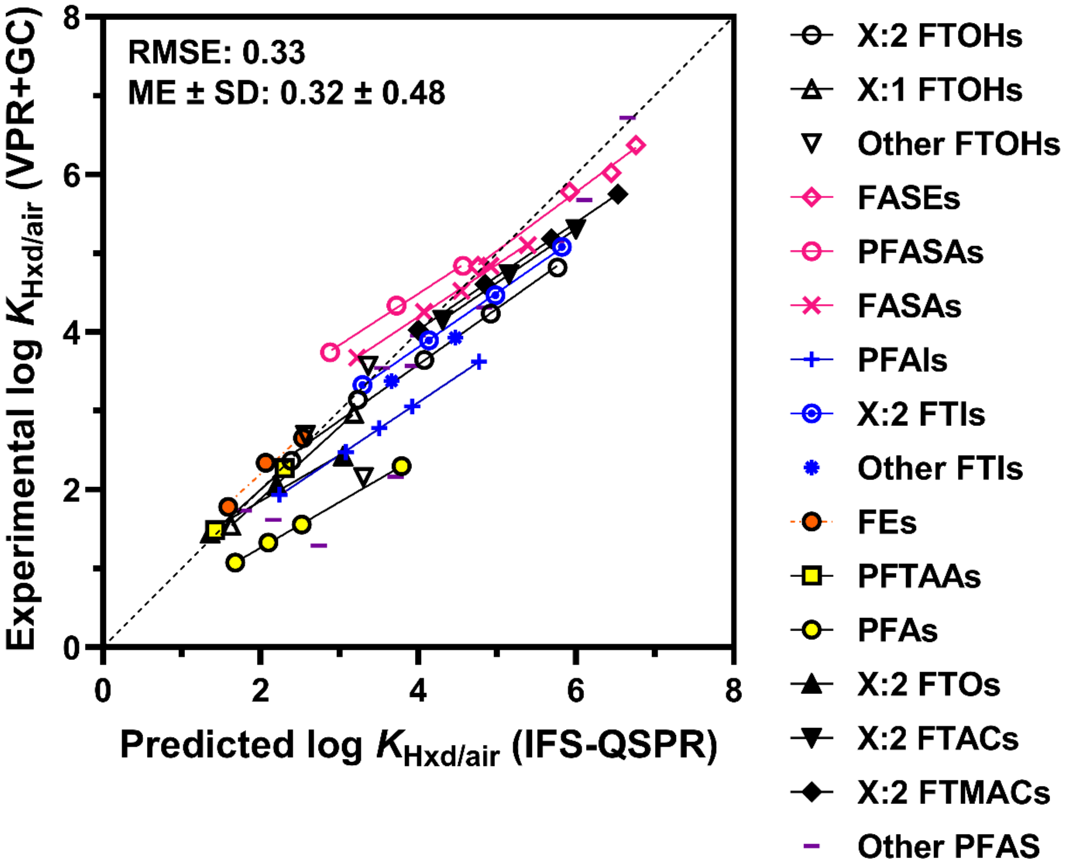

Figure S9. Experimental log $K_{Hxd/air}$ values vs predicted values by a **former version** of IFS-QSPR (implemented at the website of the UFZ-LSER database http://www.ufz.de/lserd, accessed on June 30, 2022). Experimental data used are the "recommended values" in Table 1 of the main article. RMSE, root mean squared error; ME, mean of prediction errors; SD, standard deviation of prediction errors. The solid lines indicate the linear regression for each group, and the dashed line the 1:1 relationship.

References

1. Poole, C. F.; Poole, S. K., Separation characteristics of wall-coated open-tubular columns for gas chromatography. *J. Chromatogr. A* **2008,** *1184*, (1-2), 254-280.
2. Brown, T. N., QSPRs for predicting equilibrium partitioning in solvent–air systems from the chemical structures of solutes and solvents. *J. Solution Chem.* **2022,** *51*, 1101–1132.
3. EAS-E Suite (Ver.0.95 - BETA, release Feb., 2022). www.eas-e-suite.com. Developed by ARC Arnot Research and Consulting Inc., Toronto, ON, Canada.
